# Supplementary material for: Transcript-guided targeted cell enrichment for scalable single-nucleus RNA sequencing
Source: Cell Genom. 2025 Dec 11;6(3):101101. doi: 10.1016/j.xgen.2025.101101 (PMC12985360; doi:10.1016/j.xgen.2025.101101)
Supplement: Document S1. Figures S1–S14 [file mmc1.pdf]

Cell Genomics, Volume *6*

Supplemental information

Transcript-guided targeted cell enrichment

for scalable single-nucleus RNA sequencing

Andrew Liao, Zehao Zhang, Andras Sziraki, Abdulraouf Abdulraouf, Abid Rehman, Zihan Xu, Ziyu Lu, Weirong Jiang, Alia Arya, Jasper Lee, Manolis Maragkakis, Wei Zhou, and Junyue Cao

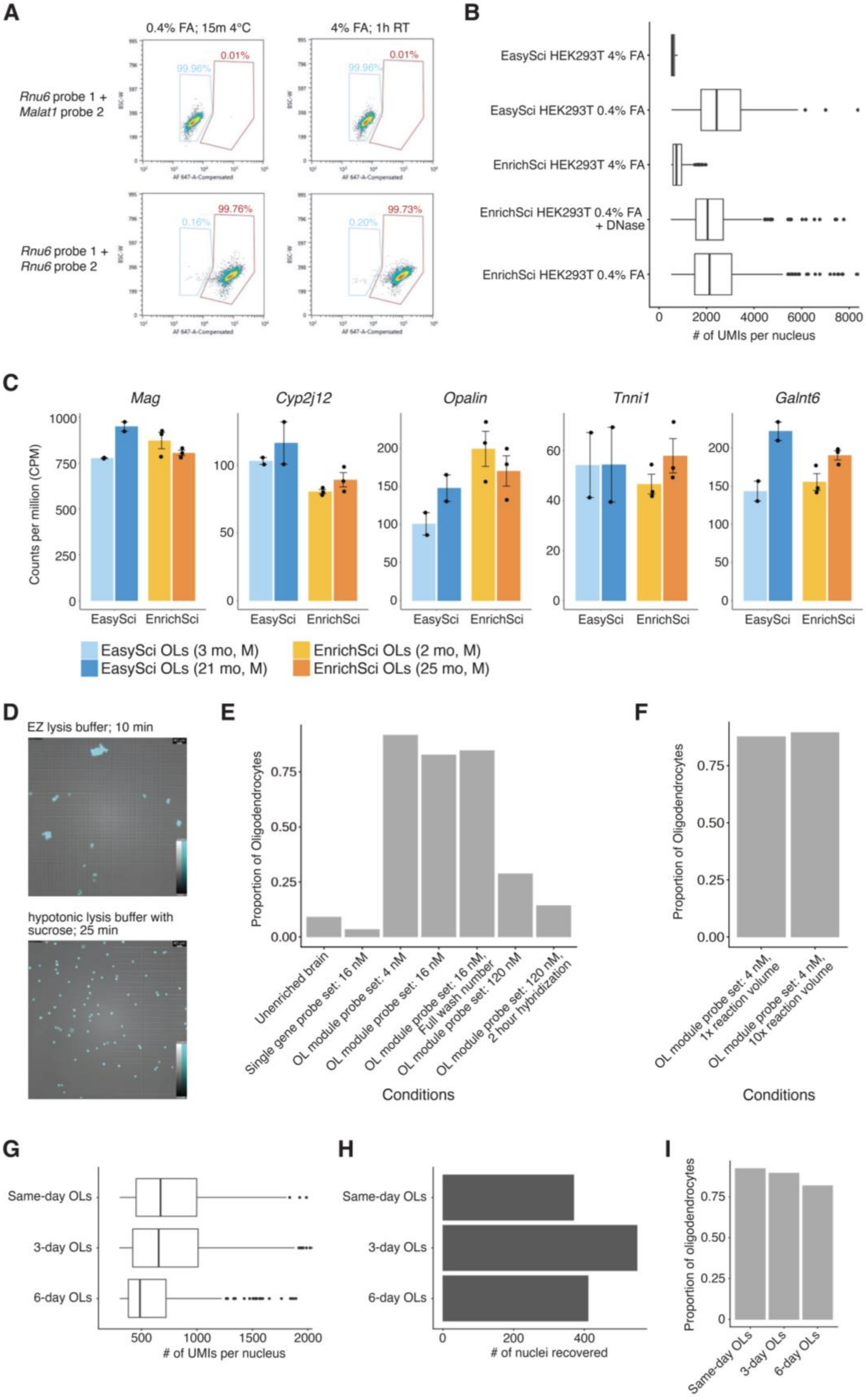

A
0.4% FA; 15m 4°C
4% FA; 1h RT
Rnu6 probe 1 + Malat1 probe 2
Rnu6 probe 1 + Rnu6 probe 2
99.96%
0.01%
0.16%
99.76%
0.20%
99.73%
B
EasySci HEK293T 4% FA
EasySci HEK293T 0.4% FA
EnrichSci HEK293T 4% FA
EnrichSci HEK293T 0.4% FA + DNase
EnrichSci HEK293T 0.4% FA
# of UMIs per nucleus
C
Mag
Cyp2j12
Opalin
Tnni1
Galnt6
Counts per million (CPM)
EasySci
EnrichSci
EasySci OLs (3 mo, M)
EasySci OLs (21 mo, M)
EnrichSci OLs (2 mo, M)
EnrichSci OLs (25 mo, M)
D
EZ lysis buffer; 10 min
hypotonic lysis buffer with sucrose; 25 min
E
Proportion of Oligodendrocytes
Unenriched brain
Single gene probe set: 16 nM
OL module probe set: 4 nM
OL module probe set: 16 nM
OL module probe set: 16 nM, Full wash number
OL module probe set: 120 nM
OL module probe set: 120 nM, 2 hour hybridization
Conditions
F
Proportion of Oligodendrocytes
OL module probe set: 4 nM, 1x reaction volume
OL module probe set: 4 nM, 10x reaction volume
Conditions
G
Same-day OLs
3-day OLs
6-day OLs
# of UMIs per nucleus
H
Same-day OLs
3-day OLs
6-day OLs
# of nuclei recovered
I
Proportion of oligodendrocytes
Same-day OLs
3-day OLs
6-day OLs

Figure S1: EnrichSci was benchmarked and optimized for robust data quality and performance, related to Figure 1. (A) Flow cytometry plots showing HCR signal in HEK293T nuclei fixed in 0.4% (left) versus 4% (right) formaldehyde. Samples were labeled using a negative control mismatched probe pair (top) or a positive control probe pair targeting *Rnu6* (bottom). **(B)** Boxplot showing unique molecular identifier (UMI) count distributions of nuclei profiled by EnrichSci and EasySci, comparing different fixation conditions and the addition of DNase treatment. **(C)** Barplots showing expression of oligodendrocyte-specific module genes (*Mag*, *Cyp2j12*, *Opalin*, *Tnni1*, *Galnt6*) in young and aged male OLs in the EasySci atlas and EnrichSci dataset. Expression is normalized to counts per million (CPM) and shown as mean ± SEM across replicates (dots). **(D)** Comparison of nuclei extraction buffers for A375 cells. Nuclei prepared in a hypotonic lysis buffer with sucrose (bottom) exhibit markedly reduced clumping and higher recovery than those processed with the commercial EZ Lysis Buffer (top). (**E**) Evaluation of HCR RNA FISH conditions for oligodendrocyte enrichment from mouse brain. We varied probe design (single-gene *Sox10* vs. multi-gene oligodendrocyte module probes), probe concentration (4, 16 or 120 nM), wash stringency (standard vs. reduced number of washes) and hybridization time (2 h vs. overnight), and assessed enrichment efficiency by single-nucleus sequencing. The optimal settings—multi-gene probe modules at 4 nM, reduced wash steps, and overnight hybridization—yielded the highest recovery of target cells. (**F**) Comparison of HCR reaction volumes (1× vs. 10×) for large-scale processing of mouse brain nuclei. Both volumes produced comparable enrichment efficiencies. (**G**) Boxplot showing UMI count distributions of nuclei from enriched samples stored at 4°C for 3 and 6 days compared with same-day processing, profiled by EnrichSci. Samples were shallowly sequenced for comparison purposes only (as part of a larger library to ~2,500 reads/nuclei, which is reflected in the low total UMI counts). (**H**) Barplot showing the number of nuclei recovered by EnrichSci from 2 wells loaded at ~666 cells/well under the same storage conditions as (G). **(I)** Barplot showing the proportion of oligodendrocytes in enriched samples under the same storage conditions.

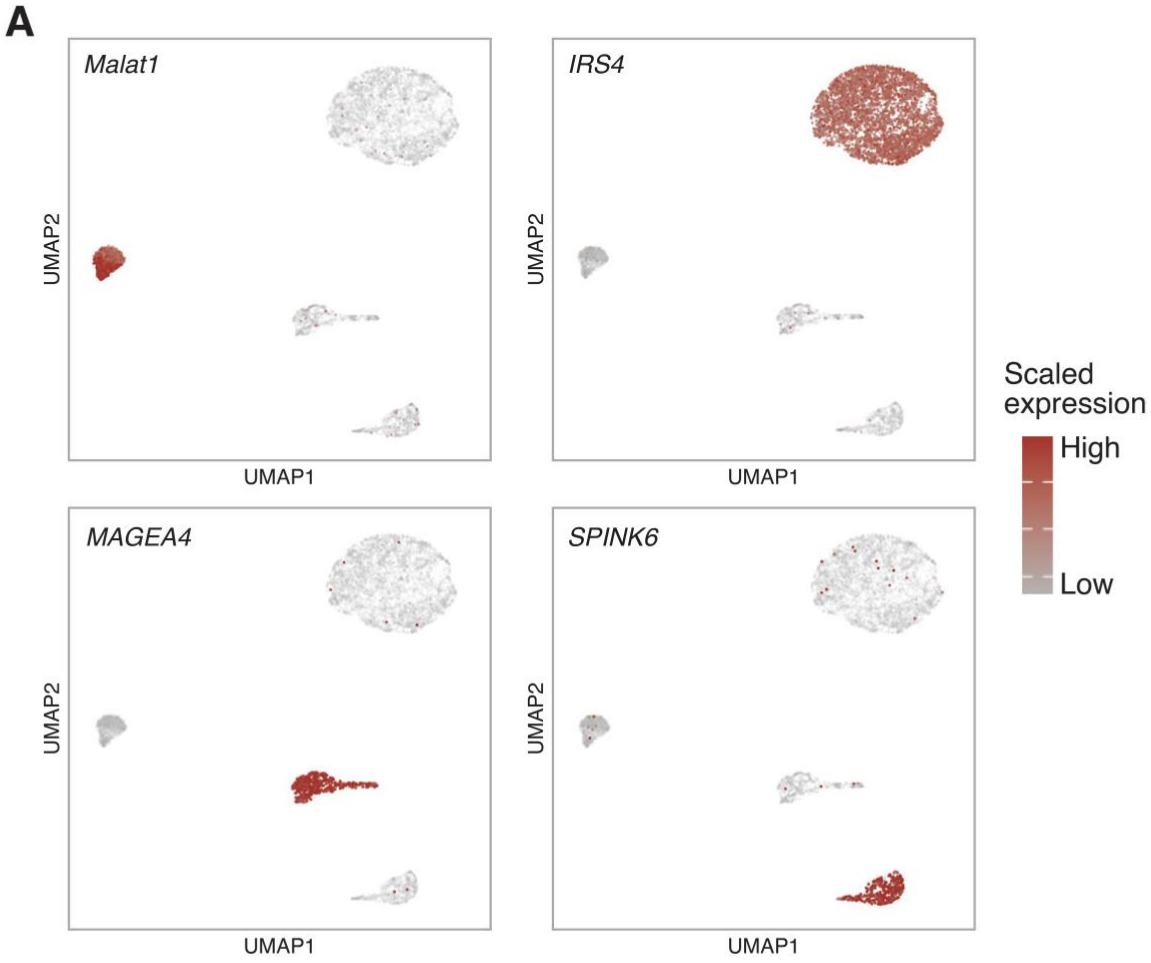

Figure S2: EnrichSci recapitulates the expected molecular states of cell lines profiled in a species-targeted experiment, related to Figure 1. (**A**) UMAP visualization of single-nucleus transcriptomes (n = 9,247) that include individual cell line spike-ins, unenriched mixture, and enriched samples profiled by EnrichSci, colored by the normalized and scaled expression of cell line-specific gene markers.

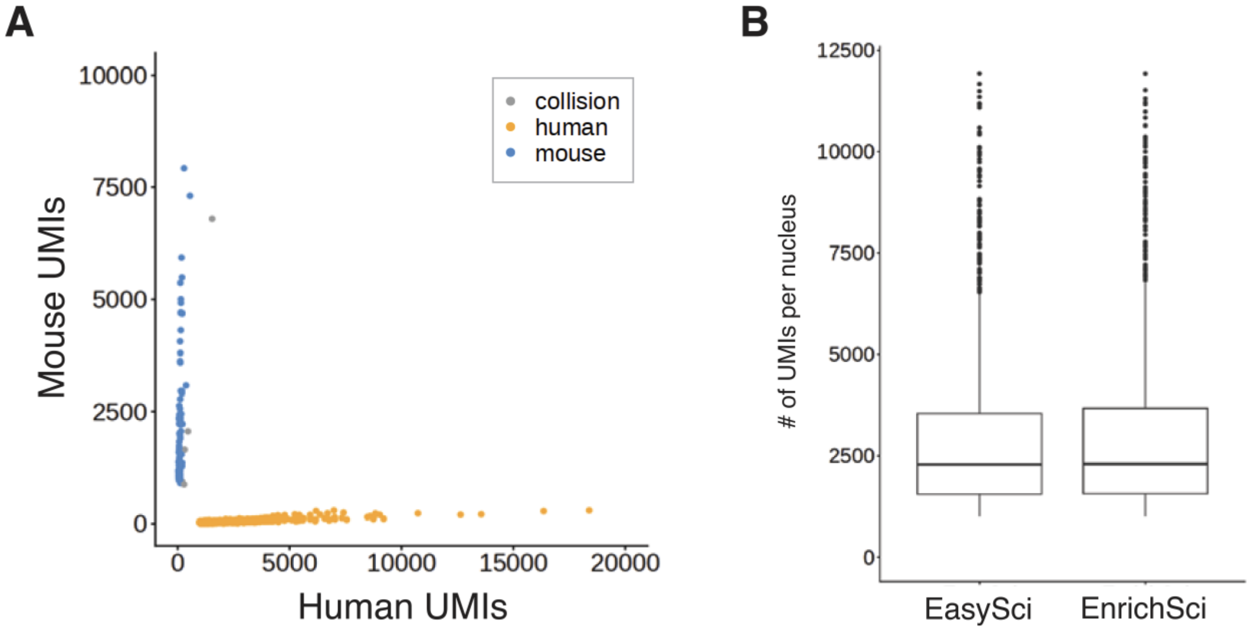

Figure S3: HCR labeling does not compromise single-cell purity or RNA capture efficiency, related to Figure 1. (**A**) Scatter plot of mouse and human UMI counts from the unenriched cell line mixture profiled by EnrichSci. Blue, inferred mouse nuclei (n = 85). Orange, inferred human nuclei (n = 814). Gray, collisions (n = 10). (**B**) Boxplot showing UMI count distribution of nuclei profiled by EasySci (n = 1,535; median = 2,296) and EnrichSci (n = 1,571; median = 2,331).

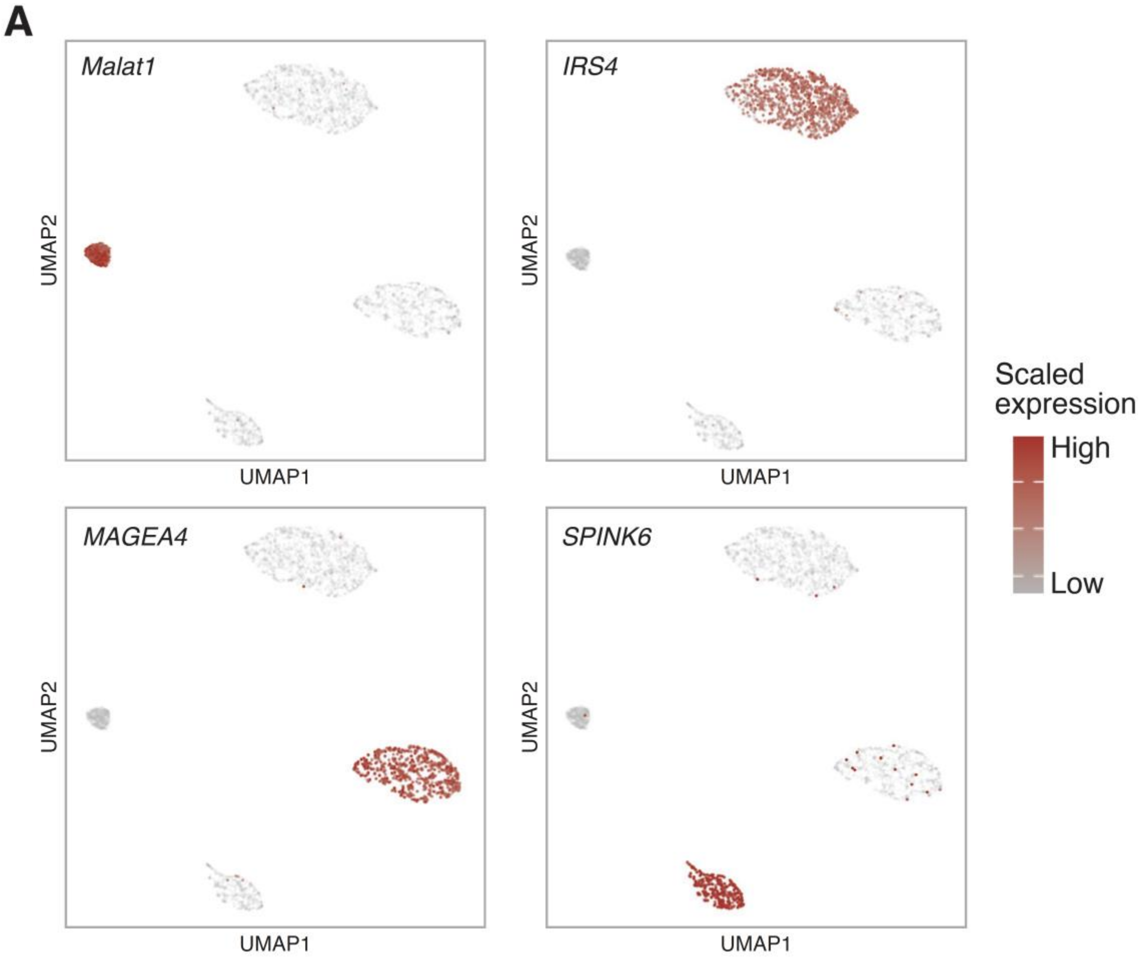

Figure S4: EnrichSci recapitulates the expected molecular states of cell lines profiled in a complex sorting experiment, related to Figure 1. (**A**) UMAP visualization of single-nucleus transcriptomes (n = 4,597) that include individual cell line spike-ins and enriched samples profiled by EnrichSci, colored by the normalized and scaled expression of cell line-specific gene markers.

A

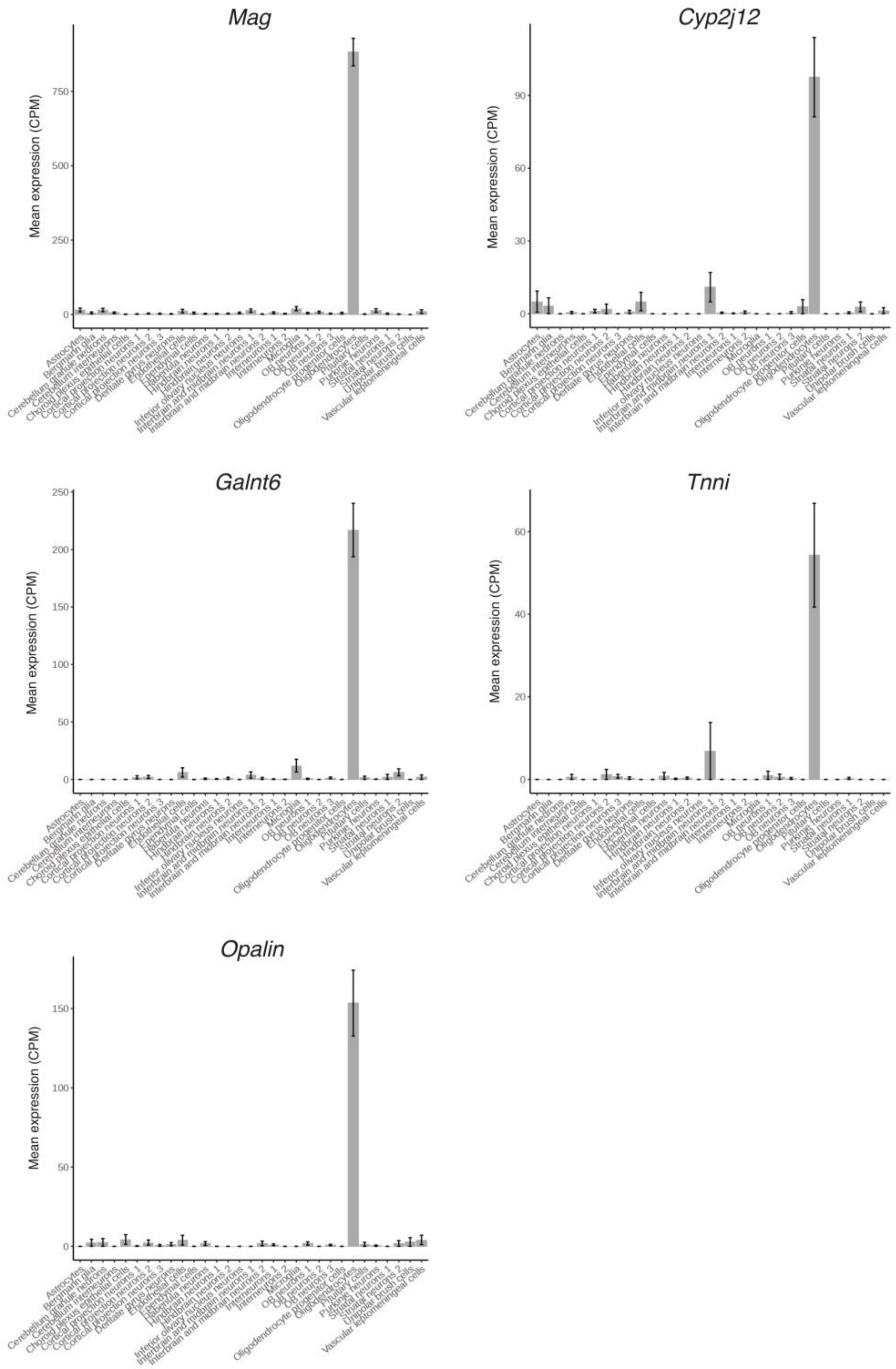

Figure S5: Oligodendrocyte-targeting genes are specifically expressed in the desired cell type, related to Figure 2. (**A**) Barplots showing expression of oligodendrocyte-specific genes across main cell types in the mouse brain.[1] Expression is normalized to counts per million (CPM) and shown as mean ± SEM across replicates.

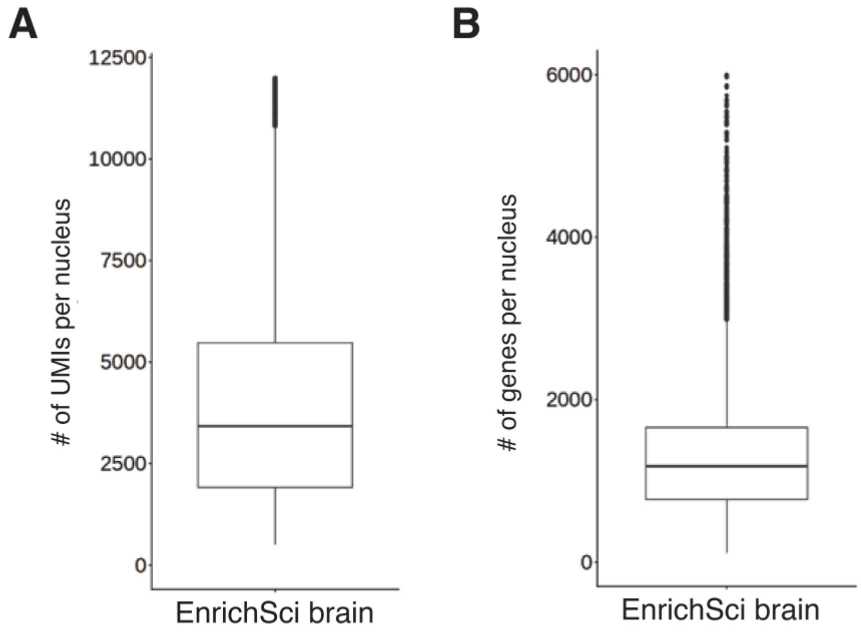

Figure S6: EnrichSci recovered single-nucleus transcriptome profiles of mouse brain nuclei with high signals, related to Figure 2. (**A**) Boxplot showing UMI count distribution of oligodendrocyte-enriched mouse brain nuclei profiled by EnrichSci. (**B**) Boxplot showing gene count distribution of oligodendrocyte-enriched mouse brain nuclei profiled by EnrichSci.

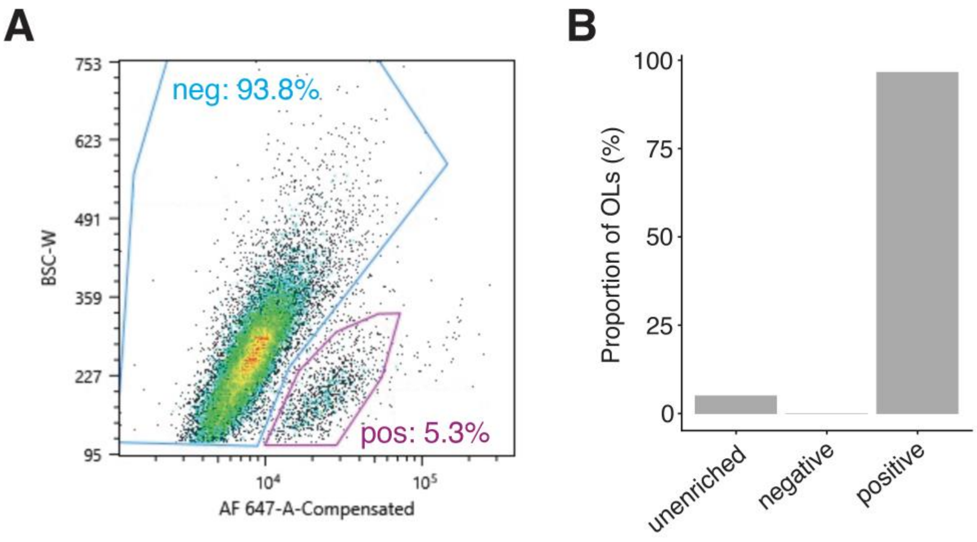

Figure S7: EnrichSci achieves enrichment and complete depletion of oligodendrocytes in positive- and negative-signal populations, related to Figure 2. (**A**) FACS plot showing positive- and negative-signal populations sorted in an oligodendrocyte-targeted experiment performed with fresh HCR reagents. (**B**) Barplot showing the proportion of oligodendrocytes in the populations sorted from (A) and profiled by EnrichSci, compared with an unenriched EasySci control.

A

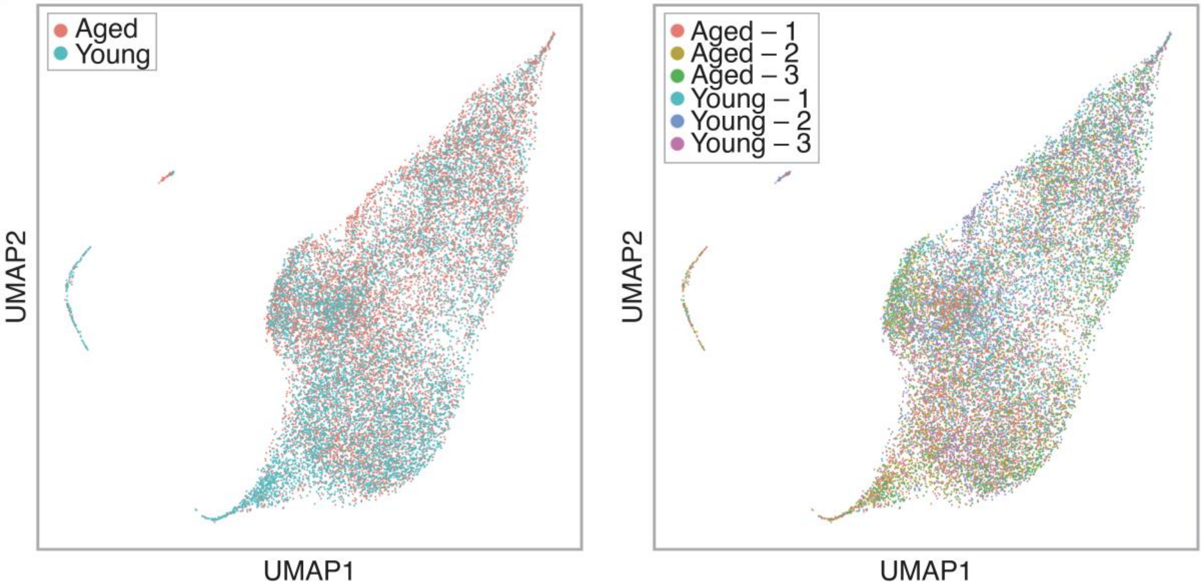

Figure S8: EnrichSci profiles oligodendrocyte nuclei with high sample consistency across age groups and replicates, related to Figure 2. (**A**) UMAP visualization of oligodendrocyte lineage nuclei (n = 18,154) profiled by EnrichSci, colored by age group (left) and individual replicate (right).

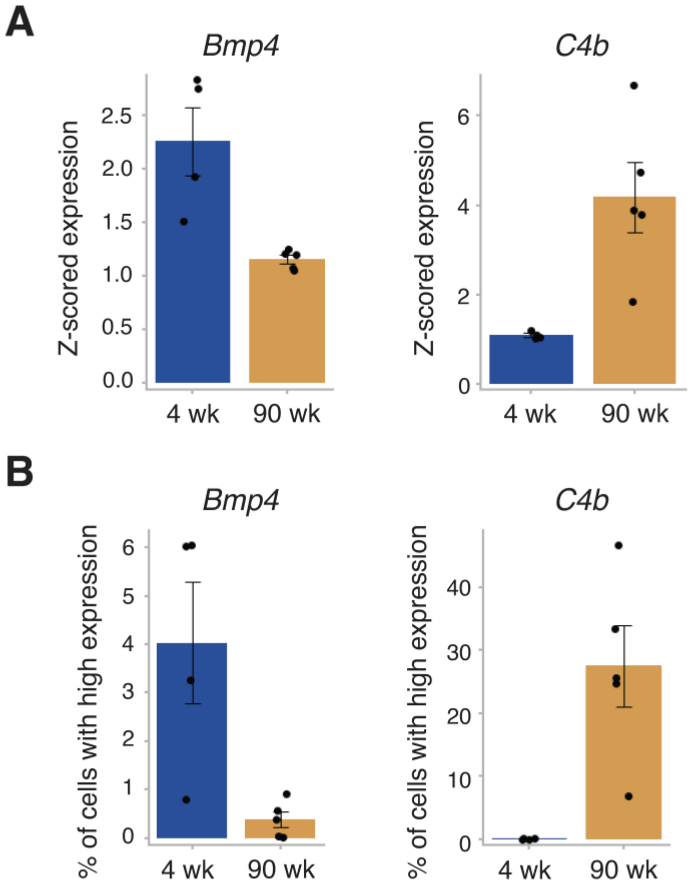

Figure S9: MERFISH confirms aging-associated oligodendrocyte subpopulation changes identified by EnrichSci, related to Figure 2. (A) Barplots showing normalized, log-transformed, and Z-scored expression of COP marker *Bmp4* (left) and reactive OL marker *C4b* (right) in OL lineage cells from young and aged mouse brains, as measured by MERFISH. Bars represent mean ± SEM across replicates (dots). **(B)** Barplots showing the fraction of cells with high expression (Z-score > 2) of *Bmp4* (left) and *C4b* (right) in each condition. Bars represent mean ± SEM across replicates (dots).

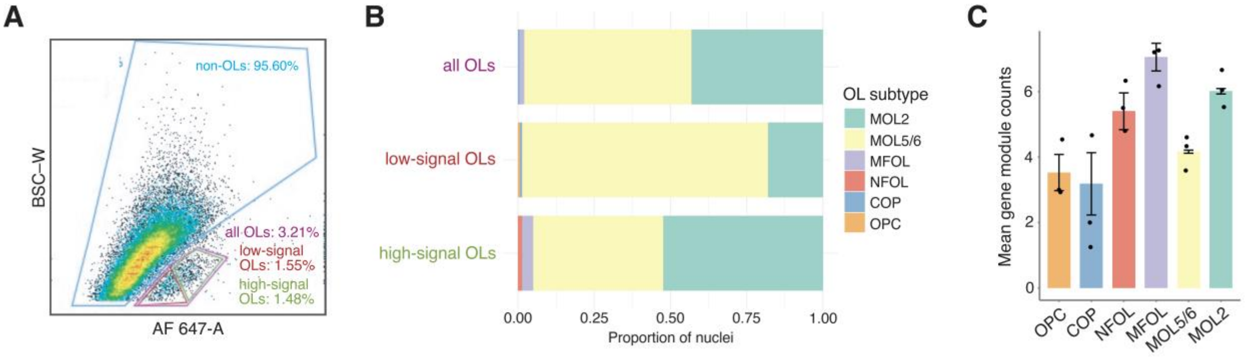

Figure S10: Low- and high-signal oligodendrocytes from complex sorting reflect subtype-specific differences in oligodendrocyte gene module expression, related to Figure 2. (A) FACS plot showing gating of low-signal, high-signal, and all OL populations during complex sorting of OL-enriched aged mouse brain nuclei. **(B)** Barplots showing the OL subtype composition of each population during complex sorting, with bars divided by subtype. **(C)** Barplots showing expression (non-normalized raw counts) of the oligodendrocyte gene module across OL subtypes in the original EnrichSci aged OL data. Bars represent mean ± SEM across replicates (dots).

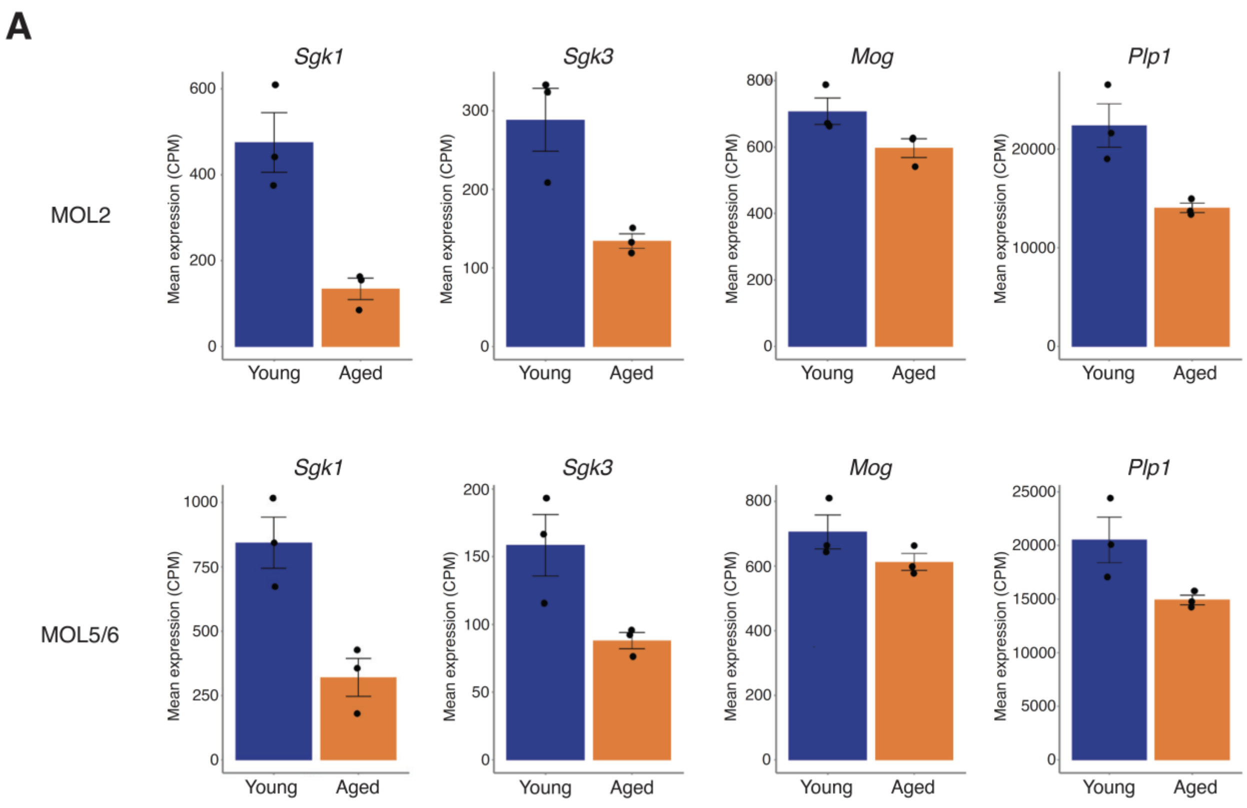

Figure S11: Mature oligodendrocyte subtypes exhibit decreased expression of key oligodendrocyte genes with age, related to Figure 3. (**A**) Barplots showing expression of *Sgk1*, *Sgk3*, *Mog*, and *Plp1* in young and aged MOL2 (top) and MOL5/6 (bottom). Expression is normalized to counts per million (CPM) and shown as mean ± SEM across replicates (dots).

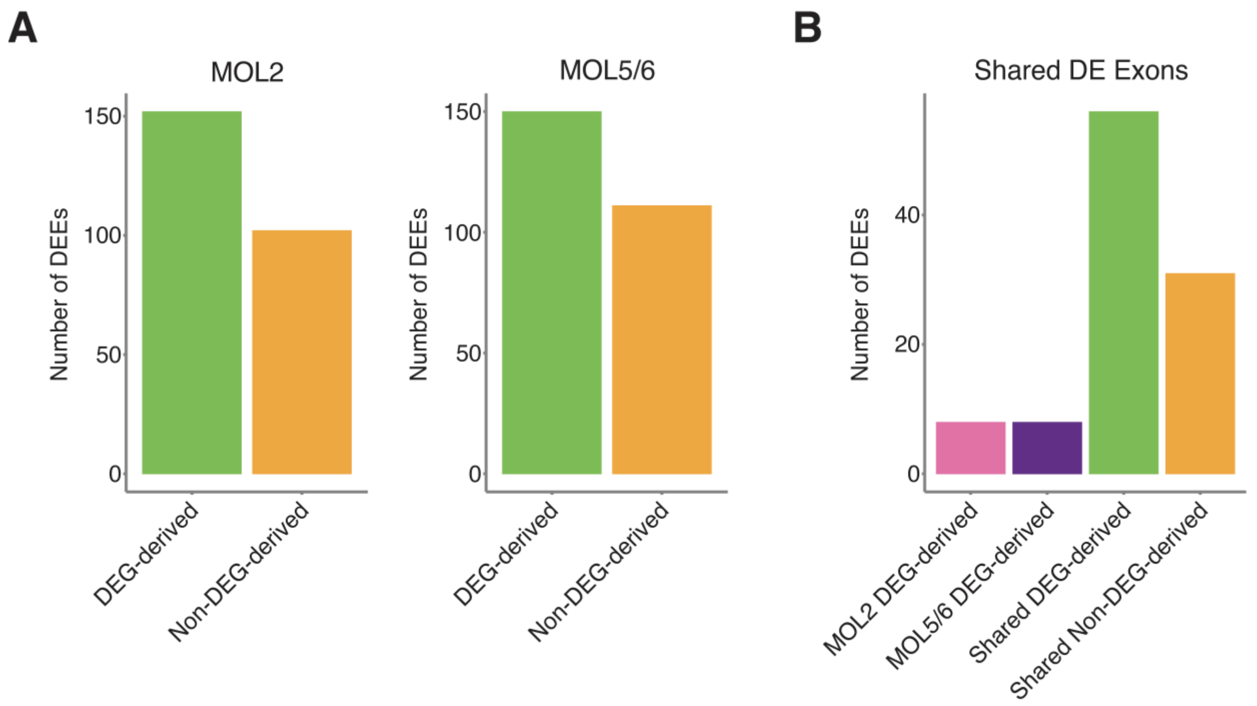

Figure S12: EnrichSci identifies substantial numbers of differentially expressed (DE) exons derived from genes that are not DE, related to Figure 3. (**A**) Barplots showing the number of DE exons (DEEs) that derive from DE genes (DEGs) and non-DEGs in MOL2 (left) and MOL5/6 (right). (**B**) Barplot showing the number of shared DEEs that derive from subtype-specific DEGs, shared DEGs, and shared non-DEGs.

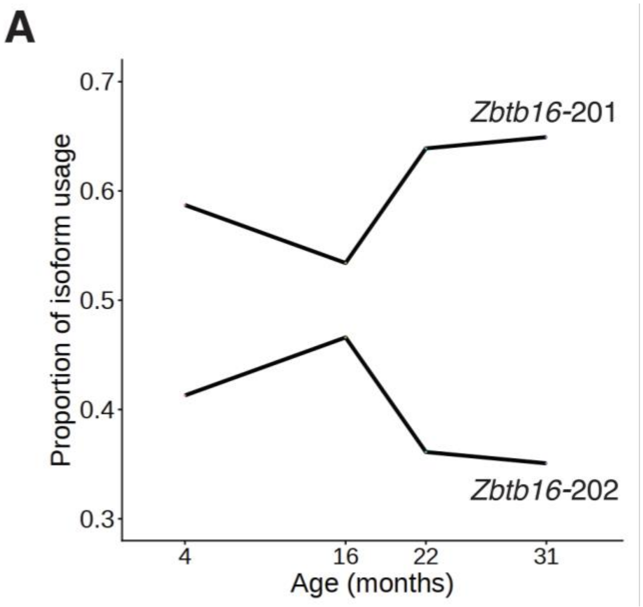

Figure S13: Long-read scRNA-seq identifies age-related shifts in *Zbtb16* isoform usage matching exon 7 changes detected by EnrichSci, related to Figure 3. (**A**) Line plot of isoform expression for *Zbtb16* in male hippocampal oligodendrocytes at 4 ages across the mouse lifespan.

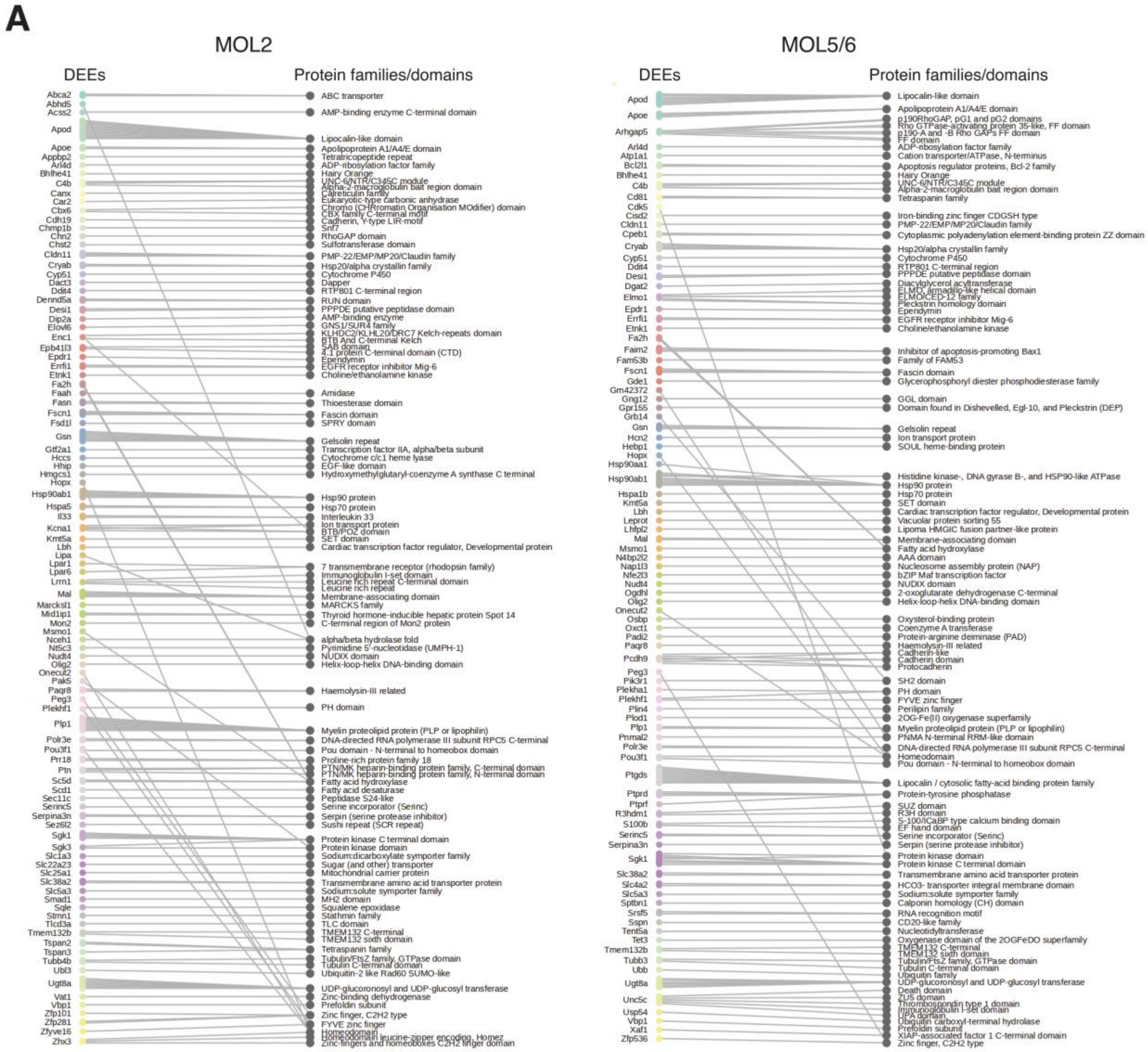

Figure S14: Differentially expressed exons code for diverse functional domains, related to Figure 3. (**A**) Bipartite visualization of DEEs and their mapped protein domains. Each point on the left represents a DEE, grouped and color-coded by parent gene, that maps to at least one annotated Pfam domain. Exons without annotated domains are excluded. Lines connect DEEs to their corresponding protein domains on the right.
