# Supplementary material for: Transcript-guided targeted cell enrichment for scalable single-nucleus RNA sequencing
Source: Cell Genom. 2025 Dec 11;6(3):101101. doi: 10.1016/j.xgen.2025.101101 (PMC12985360; doi:10.1016/j.xgen.2025.101101)
Supplement: Document S2. Article plus supplemental information [file mmc5.pdf]

Cell Genomics

Short article

Transcript-guided targeted cell enrichment for scalable single-nucleus RNA sequencing

Graphical abstract

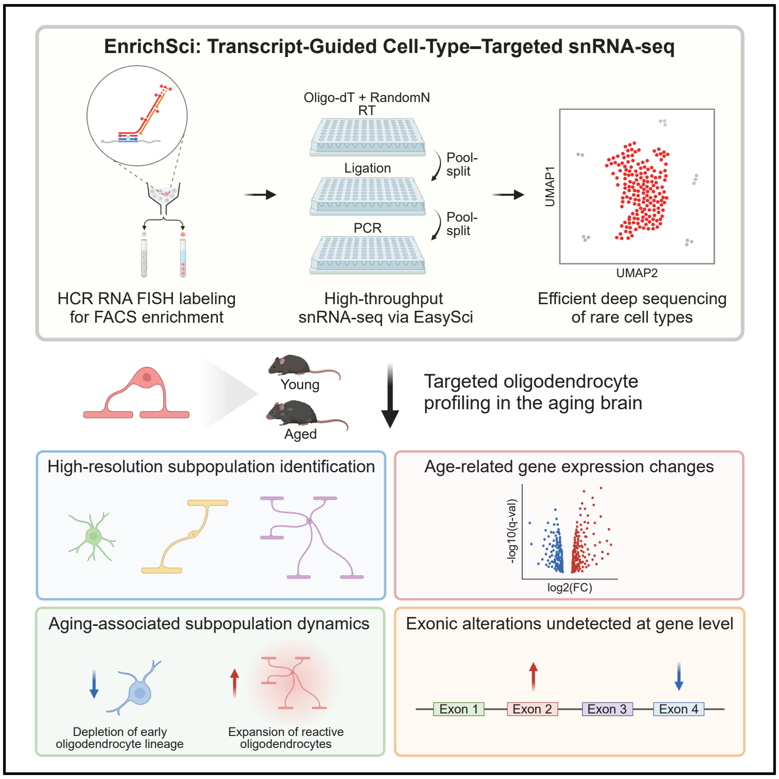

Highlights

- EnrichSci enables antibody-free, targeted snRNA-seq of rare cell types
- Efficient profiling captured age-related shifts in rare oligodendrocyte subtypes
- EnrichSci detected gene- and exon-level expression changes in aged oligodendrocytes
- Exon dynamics are linked to isoform switching and splicing factor dysregulation

Authors

Andrew Liao, Zehao Zhang, Andras Sziraki, ..., Manolis Maragkakis, Wei Zhou, Junyue Cao

Correspondence

wzhou@rockefeller.edu (W.Z.), jcao@rockefeller.edu (J.C.)

In brief

EnrichSci is an antibody-free, targeted single-nucleus RNA sequencing method that enables efficient profiling of rare cell types with full gene-body coverage. Applied to oligodendrocytes in the brain, it uncovers aging-associated gene expression changes as well as exon-level dynamics that are linked to isoform switching and splicing factor dysregulation.

Liao et al., 2026, Cell Genomics 6, 101101
March 11, 2026 © 2025 The Authors. Published by Elsevier Inc.
https://doi.org/10.1016/j.xgen.2025.101101

CellPress

Cell Genomics

CellPress
OPEN ACCESS

Short article

Transcript-guided targeted cell enrichment for scalable single-nucleus RNA sequencing

Andrew Liao,[1,2] Zehao Zhang,[1,3] Andras Sziraki,[1,3] Abdulraouf Abdulraouf,[1,2] Abid Rehman,[4,5] Zihan Xu,[1,3] Ziyu Lu,[1,3] Weirong Jiang,[1] Alia Arya,[1] Jasper Lee,[1] Manolis Maragkakis,[4] Wei Zhou,[1,6,*] and Junyue Cao[1,6,7,*]
[1]Laboratory of Single-Cell Genomics and Population Dynamics, the Rockefeller University, New York, NY, USA
[2]The Tri-Institutional MD-PhD Program, New York, NY, USA
[3]The David Rockefeller Graduate Program in Bioscience, the Rockefeller University, New York, NY, USA
[4]Laboratory of Genetics and Genomics, National Institute on Aging, Intramural Research Program, National Institutes of Health, Baltimore, MD, USA
[5]Bioinformatics and Systems Biology Program, University of California, San Diego, La Jolla, CA, USA
[6]Senior author
[7]Lead contact
*Correspondence: wzhou@rockefeller.edu (W.Z.), jcao@rockefeller.edu (J.C.)
https://doi.org/10.1016/j.xgen.2025.101101

SUMMARY

Large-scale single-cell atlases have revealed many aging- and disease-associated cell types, yet these populations are often underrepresented in heterogeneous tissues, limiting detailed molecular analyses. To address this, we developed EnrichSci—a scalable, microfluidics-free platform that combines hybridization chain reaction RNA fluorescence *in situ* hybridization (FISH) with combinatorial indexing to profile single-nucleus transcriptomes of target cell types with full gene-body coverage. Applied to oligodendrocytes in the aging mouse brain, EnrichSci uncovered aging-associated molecular dynamics across distinct oligodendrocyte subtypes, revealing both shared and subtype-specific gene expression changes. Additionally, we identified aging-associated exon-level signatures missed by conventional gene-level analyses, highlighting post-transcriptional regulation as a critical dimension of cell-state dynamics in aging. By coupling transcript-guided enrichment with a scalable sequencing workflow, EnrichSci provides a versatile approach to decode dynamic regulatory landscapes in diverse cell types from complex tissues.

INTRODUCTION

Mammalian organs are maintained through homeostasis of hundreds to thousands of distinct cell states, from common populations, such as hepatocytes (~70% of liver cells), to rare types, such as pinealocytes (<0.01% of brain cells).[1,2] Although large-scale single-cell genomics studies have cataloged many such populations, rare cell types are left underrepresented, limiting detailed characterization of their molecular heterogeneity and dynamics and complicating the development of targeted therapeutic interventions.

Recently, several groups developed methods coupling transcript-based enrichment of rare cell types with downstream transcriptome analysis.[3–5] These techniques bypass the cost and technical constraints of antibody-based enrichment, though each faces limitations. Probe-seq[3] first integrated RNA fluorescence *in situ* hybridization (FISH) with downstream bulk RNA sequencing (RNA-seq) to enable antibody-free targeted profiling of specific cell types, inspiring the development of analogous approaches with single-cell resolution. FIND-seq[4] achieved transcript-guided, cell-type-targeted single-cell RNA-seq (scRNA-seq) by coupling PCR-based transcript detection with microfluidic cytometry, but it has limited throughput (~10^3 cells) and requires specialized equipment. Most recently, PERFF-seq[5] effectively combined RNA FISH-based fluorescence-activated cell sorting (FACS) with 10× Flex, a commercial scRNA-seq platform with higher throughput (~10^5 cells). However, Flex utilizes pre-defined probe sets to capture a limited transcriptomic view, hindering the analysis of different species samples as well as the dynamics of genome-wide RNA elements (e.g., exons, introns, and many noncoding genes) crucial for cell-state regulation.

To overcome these limitations, we developed EnrichSci—a highly scalable single-cell combinatorial indexing (sci) approach[6] designed for genome-wide analysis of gene and exon expression in enriched cell populations. EnrichSci combines our EasySci single-cell platform,[1] which can process tens of millions of cells per study[2] at <$0.001/cell and supports cellular and nuclear fixation, with a hybridization chain reaction (HCR) RNA FISH workflow.[7,8] This integration enables targeted enrichment and efficient deep sequencing of rare cell types from complex tissues, resulting in detailed analyses of subtype-specific transcriptional signatures and dynamics with exon-level resolution.

RESULTS

The EnrichSci workflow (Figure 1A) begins by analyzing existing single-nucleus RNA-seq (snRNA-seq) data to select a module of

This is an open access article under the CC BY license (http://creativecommons.org/licenses/by/4.0/).

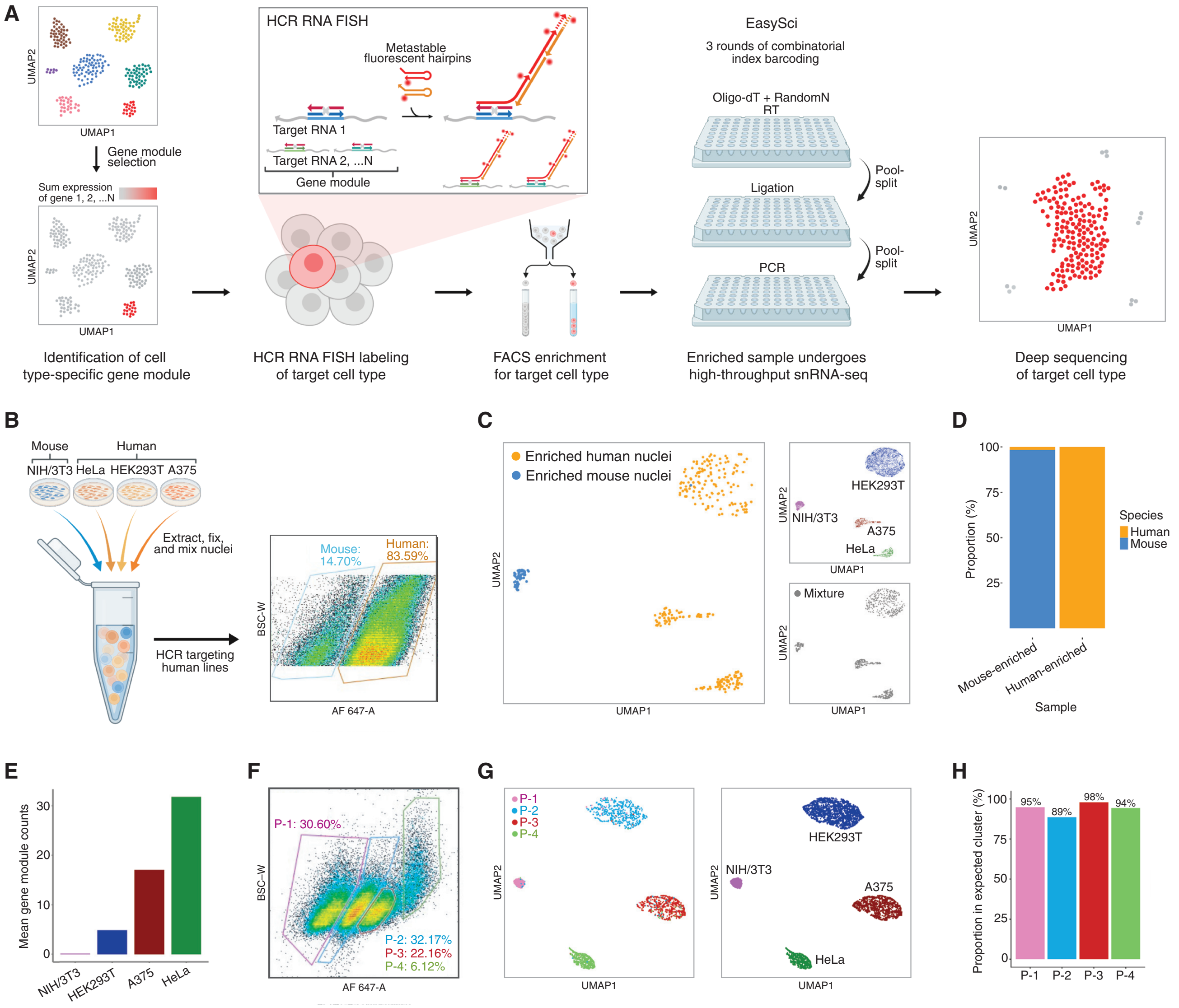

Figure 1. EnrichSci applies transcript-guided cell enrichment for targeted profiling and demonstrates proof of concept *in vitro*

(A) Schematic of the EnrichSci workflow.

(B) Design of the cell line mixture experiment (left) and FACS plot showing species-enriched populations obtained from the mixture (right).

(C) UMAP visualization of nuclei from species-enriched populations ($n = 341$, left), spike-in cell line controls ($n = 7{,}997$, top right), and unenriched mixture ($n = 909$, bottom right).

(D) Barplot showing the proportion of nuclei from different species in mouse- and human-enriched samples.

(E) Barplot showing expression of the human-specific gene module across nuclei from the 4 cell lines. Non-normalized mean counts are shown, as the HCR signal is not normalized to total gene expression within nuclei.

(F) FACS plot showing four populations complex-sorted from a cell line mixture labeled via HCR using the gene module in (E).

(G) UMAP visualization of complex-sorted nuclei ($n = 2{,}207$, left) profiled by EnrichSci, colored by sorted population, and all profiled nuclei ($n = 4{,}597$, right), colored by cell line clusters as defined by individual cell line controls and marker expression.

(H) Barplot showing the proportion of nuclei from each sorted population that correctly clustered with its expected cell line, based on ordered expression of the gene module.

5–10 genes specific to the target cell type. Next, nuclei are extracted from complex tissues or cell lines, fixed in formaldehyde, and fluorescently labeled using HCR RNA FISH[7,8] before enrichment via FACS and processing through a modified EasySci snRNA-seq workflow (STAR Methods). By employing both oligo-dT and random hexamer primers during reverse transcription, EnrichSci delivers full gene-body coverage, including introns and exons, which require deep sequencing for effective resolution. Our plate-based combinatorial indexing approach also avoids reliance on microfluidic systems required by other methods[4,5] and offers scalable parallel processing of many samples via unique first-round barcodes.

Development and benchmarking of EnrichSci

Like previous sci-RNA-seq approaches,[6,9] EasySci is compatible with cell and nuclei fixation, a required step for HCR RNA FISH. In developing EnrichSci, we therefore anticipated that HCR labeling could be coupled with EasySci profiling and were pleasantly surprised to find the two protocols were readily compatible. To initially couple the workflows, we directly performed EasySci following HCR and FACS isolation of nuclei fixed under various conditions. The Molecular Instruments (MI) HCR protocol recommends whole-cell fixation in 4% formaldehyde for 1 h at room temperature; however, because our workflow applies HCR to nuclei, we also tested milder fixation conditions, as harsher crosslinking reduces unique molecular identifier (UMI) recovery via EasySci.[1] We found that gentle fixation (0.4% formaldehyde for 15 min at 4°C) maximized UMI counts with a minimal impact on HCR signal to noise, whereas harsher fixation significantly reduced transcript capture (Figures S1A and S1B). Notably, we also compared unenriched EnrichSci libraries to EasySci libraries generated from the same fixed nuclei sample and observed comparable UMI yields (Figure S1B). This is expected, as sci-RNA-seq[6,9] has been shown to be readily compatible with formaldehyde fixation, which is commonly used in RNA FISH workflows. Additionally, we hypothesize that the formamide incubations during the HCR protocol may actually boost the RNA-seq signal recovered, as formamide has been shown to suppress RNase activity[10] and denature RNA secondary structures.[11]

In addition to benchmarking fixation conditions, we performed several checks to ensure the robustness of EnrichSci data. First, we applied a DNase treatment following HCR and FACS, as described in the PERFF-seq protocol,[5] which reported that the removal of the HCR polymer was necessary for UMI recovery via 10× Flex. In contrast, DNase treatment did not affect UMIs recovered via EnrichSci (Figure S1B), and we therefore excluded this step from our protocol. We also verified that expression of HCR-targeted genes in enriched samples profiled by EnrichSci was comparable to their expression in unenriched samples profiled by EasySci (Figure S1C), indicating that reverse transcription was not inhibited by the presence of HCR probes or polymers. We hypothesize that this robustness may reflect the strong strand displacement capabilities of commercial MMLV-derived RNase H Minus reverse transcriptase enzymes.[12] Moreover, we used a relatively low number of ~10 probe pairs per target gene (Table S1), further minimizing the potential for interference with cDNA synthesis.

To further improve EnrichSci's performance, we optimized multiple steps across the nuclei extraction, HCR, and snRNA-seq library preparation protocols (STAR Methods). Extracting nuclei using a hypotonic lysis buffer with added sucrose resulted in cleaner nuclei extraction with less debris than the commercial EZ lysis buffer (Figure S1D), which was critical for downstream HCR, as incomplete lysis and debris led to severe nuclei clumping; extending the lysis time also improved the quality of the nuclei preparation. With this optimized nuclei extraction, we next refined the HCR protocol, which had not previously been applied to isolated nuclei until the development of PERFF-seq.[5] First, we applied HCR using probes targeting a module of 5–10 genes, which improved labeling specificity over a traditional single-transcript approach (Figure S1E). Additionally, we found that HCR probe concentrations for nuclei were especially sensitive for optimal target enrichment, as increasing the individual probe concentration of an oligodendrocyte (OL)-specific gene module from 4 nM to even just 16 nM—within the range suggested by the MI whole-cell HCR protocol—caused off-target effects that obscured oligodendrocyte enrichment with noise from abundant cell types (Figure S1E). Next, because the HCR workflow loses substantial numbers of nuclei from extensive washing, we minimized wash steps while increasing wash volumes, which reduced nuclei loss and maintained HCR efficiency (Figure S1E). We also scaled the HCR hybridization reaction volume up to 10-fold to enable sufficient nuclei recovery for large-scale profiling and likewise observed no loss of HCR efficiency (Figure S1F). Finally, the EnrichSci snRNA-seq library preparation was largely unchanged from the EasySci protocol except that after FACS, we used the sorted nuclei suspension directly for reverse transcription. This avoided an additional spin down with supernatant removal, which we found to cause nuclei loss and inconsistent concentrations when working with small numbers of nuclei. For all major experiments in this study, reverse transcription through second-strand synthesis was performed immediately after FACS; however, as noted in the PERFF-seq protocol,[5] we have found that samples can be stored at 4°C for up to 6 days post-FACS while still yielding usable data, demonstrating the robustness of the workflow to delayed processing (Figures S1G–S1I).

In vitro validation of EnrichSci using mixed cell lines

As a proof of concept for enriched cell profiling, we extracted and fixed nuclei from mouse NIH/3T3 cells and human HeLa, HEK293T, and A375 cells, mixed them to simulate a heterogeneous population (Figure 1B), and performed HCR using probes against human-specific transcripts (*SCHLAP1*, *LIMCH1*, *PTMS*, *PDE4D*, *CAST*, *PDE3A*, *TPM4*, *IQGAP1*, *AKT3*, *RBMS3*, *SERPINE2*, *WWTR1*, and *FTH1*) (Table S1). In this first experiment, we separated the mixture by species into fluorescent-positive (human) and fluorescent-negative (mouse) nuclei (Figure 1B). EnrichSci profiling recapitulated the expected molecular states of each cell line (Figures 1C and S2), with nearly all enriched nuclei correctly clustering with their expected species (Figure 1D). We also confirmed that HCR labeling did not compromise downstream single-cell purity or RNA capture efficiency (Figure S3).

Next, we applied a more complex sorting strategy to the mixture, with the goal of separating all four cell lines individually. For this purpose, the aforementioned human-specific module was designed with differential, progressively increasing expression across NIH/3T3, HEK293T, A375, and HeLa nuclei (Figure 1E). The initial species-targeted experiment lacked sufficient signal to noise to achieve this resolution, but after optimizing the nuclei HCR workflow, labeling the mixture clearly separated four populations (Figure 1F). FACS isolation and profiling of these four populations again recapitulated the expected molecular states (Figures 1G and S4), with each population accurately clustering with its corresponding cell line controls (Figure 1H).

In vivo application of EnrichSci for targeted oligodendrocyte profiling

Building on our cell line validation, we next applied EnrichSci *in vivo* by targeting mouse brain oligodendrocytes—glial cells critically involved in myelination and vulnerable to age-related degeneration.[13] We isolated brain nuclei from six male mice (three 2-month-old and three 25-month-old mice; Figure 2A) and performed HCR, targeting five oligodendrocyte-specific transcripts (*Mag*, *Galnt6*, *Opalin*, *Cyp2j12*, and *Tnni1*; Figure S5; Table S1). Following library preparation and sequencing of the oligodendrocyte-enriched samples, low-quality cells and doublets were removed (STAR Methods), yielding 19,492 high-quality nuclei with a median of 3,566 unique transcripts (1,184 genes) per nucleus (Figures 2B and S6). Cell identities were first annotated using the Allen Brain Cell Atlas MapMyCells tool[14] and confirmed by robust expression of oligodendrocyte markers *Mag* and *Plp1* (Figure 2B). Compared with unbiased single-nucleus whole-brain analysis that identified ∼5.7% oligodendrocytes,[1] our EnrichSci approach yielded ∼93% oligodendrocytes—a 16-fold enrichment (Figure 2C). An additional experiment profiling both HCR-positive and HCR-negative nuclei further validated the approach: oligodendrocytes comprised 96% and 0% of each fraction, respectively (Figure S7).

High-resolution profiling of oligodendrocyte subpopulations and their age-related dynamics

To characterize distinct oligodendrocyte subtypes, we subsetted 18,154 cells annotated as oligodendrocyte precursor cells (OPCs) or oligodendrocytes and integrated data from different ages using Seurat[15] (Figure S8). Uniform manifold approximation and projection (UMAP) visualization revealed cellular states spanning the full oligodendrogenesis spectrum—OPCs (*Pdgfra*+), committed oligodendrocyte precursors (COPs; *Bmp4*+), newly formed oligodendrocytes (NFOLs; *Prom1*+), myelin-forming oligodendrocytes (MFOLs; *Slc9a3r2*+), and mature oligodendrocytes (MOLs; *Mog*+) (Figures 2D and 2E).[16] Within the MOLs, we identified two subtypes: MOL2, marked by *Klk6* and *Hopx*, which was reported to be hindbrain specific,[17,18] and MOL5/6, which expresses *Ptgds* and *Il33* and is found across broader brain regions.[17,18] Trajectory analysis showed that MFOLs preferentially transition to MOL5/6 versus MOL2, suggesting impeded oligodendrocyte differentiation in the hindbrain (Figures 2D and 2E). This finding aligns with human single-cell studies reporting reduced oligodendrocyte differentiation in the cerebellum.[19]

To evaluate aging effects on subpopulation dynamics, we used Milo[20] to cluster cells into transcriptionally similar neighborhoods and conducted differential abundance testing between age groups. Across cell states, we observed heterogeneous aging dynamics. Intermediate precursors (COPs, NFOLs, and MFOLs) were depleted in aged brains (Figure 2F), reflecting impaired oligodendrocyte differentiation consistent with prior studies.[1,19] Meanwhile, we detected an aging-expanded MOL subpopulation marked by reactive genes (e.g., *C4b* and *Serpina3n*) (Figures 2F and 2G). These subpopulation shifts are supported by whole-brain single-cell analyses[1,18,21] as well as MERFISH data[22] that show *Bmp4*+ COP depletion and *C4b*+ reactive oligodendrocyte expansion in aged brains (Figure S9), confirming that EnrichSci can effectively dissect cellular subtypes and cell-state transitions within targeted lineages.

Finally, we applied a complex sorting strategy within the oligodendrocyte gate, subdividing it into low- and high-signal nuclei (Figure S10A). Downstream profiling revealed distinct subtype compositions: MOL5/6 predominated in the low-signal population, whereas high-signal nuclei were enriched for subtypes (NFOLs, MFOLs, and MOL2) with higher expression of the oligodendrocyte-specific module (Figures S10B and S10C).

Gene expression dynamics of MOLs subtypes in aging

Next, we examined how aging alters the transcriptomes of MOL subtypes. Differential expression (DE) analysis[23] (false discovery rate [FDR] < 0.05, fold change > 1.5) identified 251 differentially expressed genes (DEGs) in MOL2 and 294 in MOL5/6 (Figure 3A; Table S2). Despite distinct molecular and spatial profiles, both subtypes showed remarkably concordant gene expression changes in aging; the 119 DEGs identified in both subtypes showed highly correlated expression shifts (Pearson r = 0.9, p = 5e−45; Figure 3B), indicating a global transcriptional remodeling of aged MOLs. Shared upregulated genes were linked to apoptosis (e.g., *Map3k5*; *Pik3r3* and *Mapk10* in MOL2; and *Itpr1* and *Nfkb1* in MOL5/6) and Alzheimer's disease (e.g., *Apoe*, *Map3k5*, *Insr*, and *Plcb4*), whereas genes involved in heat stress response (e.g., *Hsph1*, *Hspa4l*, *Hsp90aa1*, and *Hsp90ab1*) and oligodendrocyte cell fate commitment (e.g., *Olig2*) were downregulated (Figure 3C).[24–26] We also observed reduced levels of mineralocorticoid targets *Sgk1* and *Sgk3*—key regulators of calcium channel activity and glucose uptake—and downregulation of myelination genes (e.g., *Mog* and *Plp1*) (Figure S11), matching reports of age-related myelin loss.[21] Subtype-specific alterations included downregulation of cholesterol biosynthesis (e.g., *Hmgcs1*, *Hmgcr*, *Cyp51*, *Msmo1*, *Sqle*, *Sc5d*, *Dhcr7*, and *Fdft1*) and telomere maintenance (e.g., *Zfp827*) genes in aged MOL2, alongside upregulation of genes linked to oxidative damage response (e.g., *Mapk10*) and spliceosome-mediated alternative splicing (e.g., *Nova1* and *Celf2*) (Figure 3C).[24–26] Meanwhile, aged MOL5/6 selectively downregulated genes involved in energy homeostasis (e.g., *Sorl1* and *Ubb*) and upregulated cellular senescence genes (e.g., *Itpr1*, *Atr*, *Foxo1*, and *Nfkb1*).[24–26]

Exon-level dynamics of MOL subtypes in aging

Applying the same DE framework (FDR < 0.05, fold change > 1.5) to exons, we identified 261 and 254 differentially expressed exons (DEEs) in MOL2 and MOL5/6, respectively (Figure 3D; Table S3). Of these, 103 were shared between both subtypes and exhibited tightly correlated aging dynamics (Pearson r = 0.89, p = 1.5e−142; Figure 3E). Although most DEEs mirrored the dynamics of their parent genes, a substantial fraction (42.5% of DEEs in MOL2, 40.1% in MOL5/6, and 30.1% of shared DEEs) of them were non-DEG derived (Figure S12), uncovering pathway perturbations invisible to gene-level analyses (Figure 3C).[24–26] For instance, aged MOL5/6 showed enrichment of exons in genes involved in RNA splicing regulation (e.g., *Pik3r1* and *Srsf5*) and cytokine production (e.g., *Sptbn1* and *Pik3r1*), while aged MOL2 displayed increased exon usage in neuroinflammatory response genes (e.g., *Zeb2*) and downregulation of

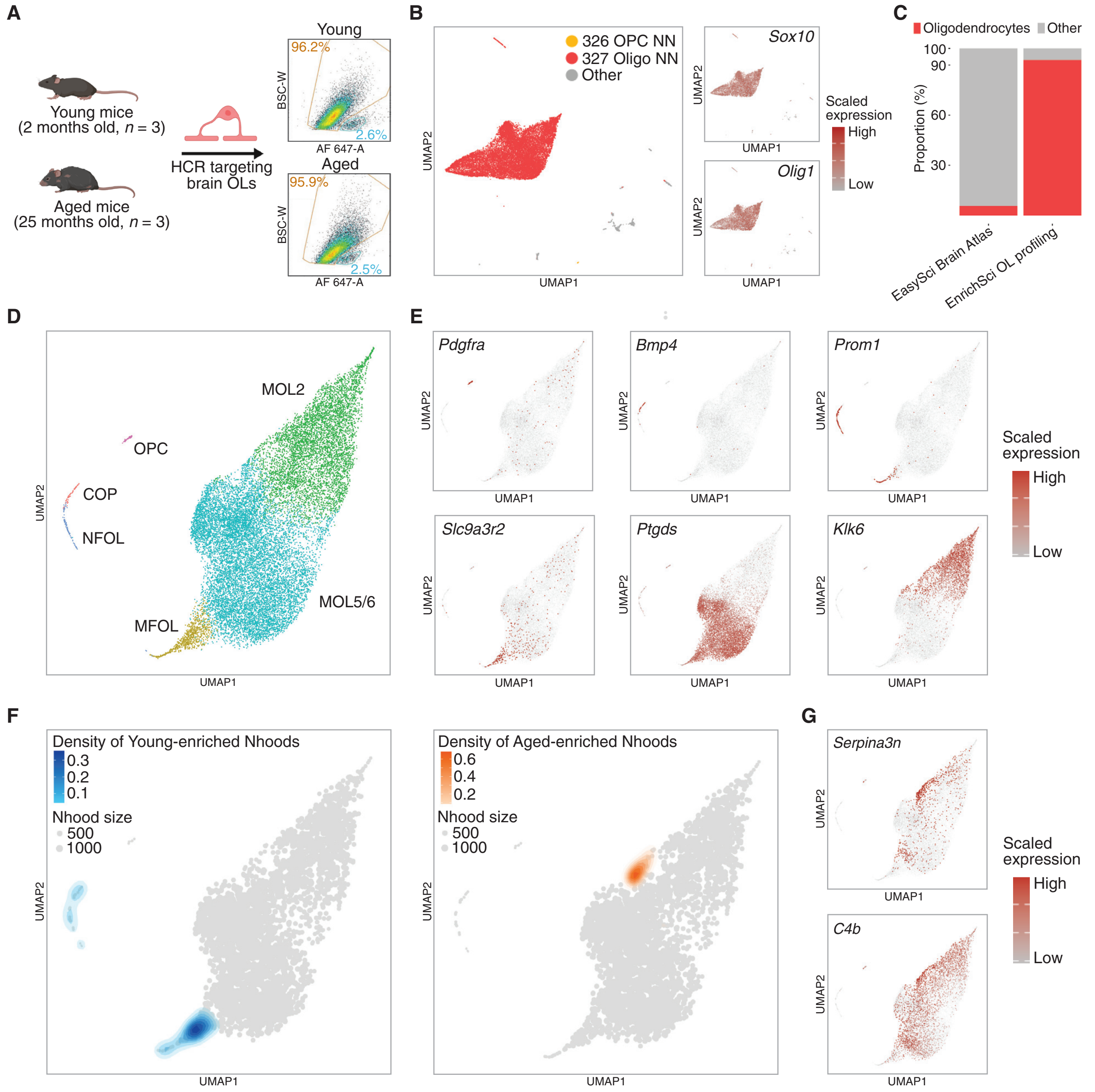

Figure 2. EnrichSci enables efficient profiling and subpopulation analysis of mouse brain oligodendrocytes

(A) Scheme showing the EnrichSci pipeline for analysis of oligodendrocytes across age groups (left) and representative FACS results (right).

(B) UMAP visualization of mouse brain nuclei (n = 19,492) profiled by EnrichSci, colored by BICCN subclass name (left) and expression of oligodendrocyte lineage markers (right).

(C) Proportion of oligodendrocytes and non-oligodendrocytes in unenriched and enriched datasets.

(D) UMAP visualization of oligodendrocyte lineage nuclei (n = 18,154) profiled by EnrichSci, colored by subtype.

(E) UMAP visualization of oligodendrocyte lineage nuclei, colored by expression of subtype-specific markers.

(F) UMAP visualization of oligodendrocyte lineage cell neighborhoods, overlaid with density of young-enriched (left) and aged-enriched (right) neighborhoods.

(G) UMAP visualization of oligodendrocyte lineage nuclei, colored by reactive oligodendrocyte markers.

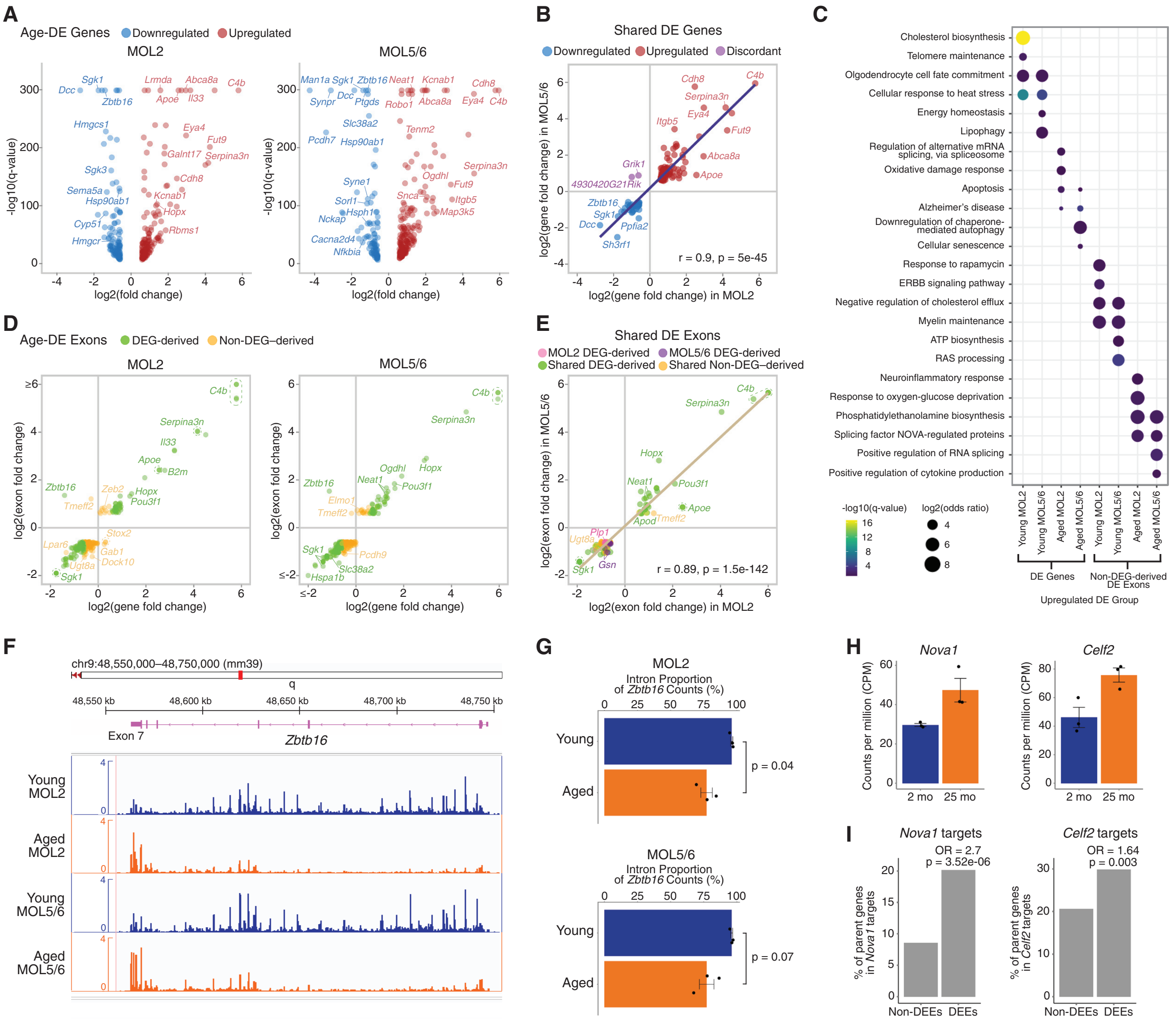

Figure 3. EnrichSci uncovers aging-associated gene- and exon-level alterations in mature oligodendrocytes

(A) Volcano plots of DEGs between young and aged MOL2 (left) and MOL5/6 (right). Downregulated genes are shown in blue and upregulated genes in red.

(B) Scatterplot showing the $\log_2$(fold change) for DEGs shared by both subtypes. Points are colored by the direction of change.

(C) Dotplot showing significantly enriched pathways for DEGs (left) and for non-DEG-derived DEEs (right). Results are stratified by subtype and age group. Dot size represents $\log_2$(odds ratio), and color indicates the $-\log_{10}(q$ value).

(D) Scatterplots of DEEs in MOL2 (left) and MOL5/6 (right). DEG-derived DEEs are shown in green and non-DEG-derived DEEs in orange.

(E) Scatterplot showing the $\log_2$(fold change) for DEEs shared by both subtypes. Points are colored by the DE status of the parent gene.

(F) Genome browser tracks showing aggregated read coverage across the *Zbtb16* gene body in young versus aged MOL2 and MOL5/6.

(G) Barplots showing the proportion of *Zbtb16* reads mapping to intronic regions in young and aged MOL2 (top) and MOL5/6 (bottom). Error bars indicate the SEM, and individual mouse replicates are overlaid as points. p values were calculated using an unpaired two-sided t test.

(H) Barplots showing MOL2 expression of DE splicing factors *Nova1* and *Celf2*. Expression is normalized to CPM and averaged across replicates. Error bars indicate the SEM.

(I) Barplots showing the proportion of non-DEEs and DEEs for MOL2 identified as targets of *Nova1* and *Celf2*. Significant enrichment of splicing factor targets was determined using Fisher's exact test.

ERBB-signaling exons linked to oligodendrocyte differentiation (Figure 3F).

Further demonstrating exon-level complexity, some DEEs showed inverse trends from their parent genes. One striking example is *Zbtb16*, a transcription factor essential for oligodendrocyte maturation whose knockout impairs social cognition and prefrontal cortex myelination.[27] Although *Zbtb16* gene expression significantly declined with age, expression of exon 7 near

CellPress
OPEN ACCESS

its 3′ end increased in aged MOL2 and MOL5/6 (Figure 3F). Validating our findings, long-read scRNA-seq[28] of the aging mouse brain found that in hippocampal oligodendrocytes, where *Zbtb16* expression was detected, usage of the exon-7-containing isoform 201 increased with age, while the exon-7-lacking isoform 202 was more abundant in young mice (Figure S13). DRIMSeq[29] pairwise isoform usage analysis confirmed a statistically significant shift in *Zbtb16* isoform usage between 4- and 31-month-old mice ($p = 0.019$). In our short-read data, we also observed an age-related shift in *Zbtb16* splicing dynamics—young MOL2 and MOL5/6 showed high intronic read fractions for *Zbtb16* (96.1% and 96.1%, respectively), which dropped to 77.1% and 77.3% in aged MOLs (Figure 3G). Together, these results highlight post-transcriptional regulation as a key layer of cell-state aging dynamics.

Functional domain mapping of DEEs

We next mapped the DEEs to their associated coding sequences, enabling annotation of their corresponding Pfam domains[30] (Figure S14; STAR Methods). In both MOL subtypes, we identified annotations including "myelin proteolipid protein" (e.g., *Plp1*) and "lipocalin-like domain" (e.g., *Apod*), suggesting direct roles in oligodendrocyte function. Many DEEs mapped to cellular-stress-associated protein families, including heat shock proteins (e.g., *Hsp90aa1*, *Hsp90ab1*, *Hspa1b*, and *Cryab*), apoptosis regulators (e.g., *Faim2* and *Bcl2l1*), and ubiquitin machinery (e.g., *Ubb* and *Usp54*). Some DEEs across different genes mapped to common functional domains, including "homeodomain" (e.g., *Hopx*, *Onecut2*, *Zhx3*, and *Pou3f1*), "SH2 domain" (e.g., *Grb14* and *Pik3r1*), "PNMA N-terminal RRM-like domain" (e.g., *Pnmal2* and *Gm42372*), and "protein kinase domain" (e.g., *Sgk3* and *Pak5*); conversely, multiple DEEs of certain genes (e.g., *C4b*) coded for different domains (e.g., exon 13, "alpha-2-macroglobulin bait region domain" and exon 41, "UNC-6/NTR/C345C module"). These results demonstrate that DEEs are not dominated by one protein family but code for diverse functional domains.

Splicing factor abundance as a driver of exon-level dynamics

To explore mechanisms underlying exon-level alterations, we examined DEEs in relation to splicing factor expression. We first filtered the DEGs for known splicing factors and then mined public CLIP-seq data[31] to identify transcripts bound by DE splicing factors. In MOL2, *Nova1* and *Celf2* gene expression increased with age (Figure 3H). Notably, MOL2 DEEs were significantly enriched for genes targeted by these splicing factors (Figure 3I), with 44 and 73 of 261 DEEs belonging to genes with *Nova1*- and *Celf2*-bound transcripts, respectively. This enrichment of DE splicing factor targets could indicate a mechanistic link between age-dependent splicing factor dysregulation and exon-level dynamics, where altered splicing factor abundance may drive changes in exon inclusion or exclusion.

DISCUSSION

Large-scale single-cell atlases have cataloged numerous aging- and disease-associated cell states,[1,2,32,33] but detailed molecular signatures for many rare populations remain poorly characterized. To address this, we introduce EnrichSci—an snRNA-seq method that leverages HCR RNA FISH for targeted cell profiling. This workflow enables flexible cell-type enrichment based on the expression of marker transcripts, including noncoding RNAs and RNAs coding for nuclear proteins. As many rare populations are distinguished by these antibody-inaccessible markers, transcript-based enrichment offers critical advantages over conventional antibody-based enrichment. Recent targeted profiling methods[3–5] have also utilized transcript-based enrichment, but EnrichSci uniquely integrates this strategy with combinatorial indexing and offers several distinct features.

First, the combinatorial indexing workflow achieves markedly greater scalability and cost efficiency than other targeted methods, both in the number of nuclei profiled (up to 10^7 nuclei)[1,2,32,33] and samples processed in parallel via unique first-round barcodes. This design enables complex sorting experiments (Figures 1E–1H and S10) that other single-cell platforms would struggle to scale. These experiments also demonstrate the potential of bespoke gene modules to distinguish many cell types. Previously, PERFF-seq[5] showed that multigene panels can enhance the HCR signal for lowly expressed transcripts; here, we expanded the approach by designing a gene module that resolved four cell types *in vitro* (Figures 1E–1H). Our oligodendrocyte-targeting module showed similar potential *in vivo*, as complex-sorted oligodendrocyte populations displayed subtype compositions consistent with their module expression (Figure S10). Further optimization of this approach, including multi-channel modules, could yield even greater ability to resolve distinct populations.

EnrichSci also uniquely offers transcriptome profiling with full gene-body coverage, capturing exon-level information critical for understanding isoform-specific and post-transcriptional regulatory events. Coupled with target enrichment, this capability is particularly powerful, as exon-level analyses necessitate deep sequencing to reliably measure such sparse data. EnrichSci thus maximizes the utility of full gene-body coverage to uncover exon-level dynamics unfeasible to interrogate via unbiased approaches.

Targeting oligodendrocytes in the aging brain, EnrichSci detected subtle age-related shifts in rare subtypes at a fraction of the cost of unbiased profiling and uncovered exon-level expression dynamics missed by traditional gene-level analyses. For example, *Zbtb16* exon 7 expression increased in aged MOLs despite a gene-level decrease in expression (Figures 3D and 3F), reflecting an isoform switch confirmed by long-read scRNA-seq[28] (Figure S13). Meanwhile, our short-read data showed reduced intronic *Zbtb16* reads in aged MOLs (Figure 3G), suggesting a shift in splicing dynamics. While these observations could be compatible with a model where altered splicing dynamics contribute to isoform switching, direct mechanistic tests (e.g., nascent RNA assays[34,35] with isoform resolution and perturbation of splicing factors) are needed to disentangle the contributions of splicing kinetics versus alternative splice-site selection in *Zbtb16* isoform usage. Nonetheless, uncovering exon-level dynamics of *Zbtb16* offers insights into this multifunctional transcription factor that regulates numerous processes (e.g., cell differentiation, immune regulation, and

apoptosis) in diverse cell types and tissues[27,36,37] and whose knockdown in mouse models of various diseases (e.g., Alzheimer's[38] and Muckle-Wells syndrome[39]) produces rescue phenotypes (e.g., improved cognitive behaviors[38] and reduced inflammatory pathogenesis[39]).

More broadly, we investigated the relationship between splicing regulation and exon-level dynamics. Binding data[31] for DE splicing factors revealed enrichment of their target genes among DEEs, suggesting that aging-associated changes in splicing factor levels may drive exon-level dysregulation. Although further validation is needed, such a model could provide new avenues to modulate age-related exon- and isoform-level changes. While many DEEs were linked to DE splicing factors, most DEEs were not identified among their targets, reflecting limitations in our analysis. Binding data were not available or sufficiently robust for all DE splicing factors, and our cutoffs may have excluded additional relevant regulators. Exon-level changes could also be driven by other RNA processing mechanisms, including alternative transcription start sites,[40] alternative polyadenylation,[41] or post-transcriptional regulation via RNA stability and degradation pathways.[42]

Altogether, these findings demonstrate the power of EnrichSci to flexibly target rare cell types and uncover molecular dynamics inaccessible to other methods. Looking ahead, we envision expanding EnrichSci to incorporate additional molecular layers (e.g., chromatin accessibility[43] and DNA methylation[44]) and genetic perturbations (e.g., CRISPR[35]), providing a versatile platform to dissect molecular dynamics and identify genetic drivers in aging- and disease-associated cell populations.

Limitations of the study

As EnrichSci utilizes prior snRNA-seq data to design cell-type-specific gene modules, its applicability in poorly characterized systems is limited. Like other HCR-based methods,[5] EnrichSci also requires large nuclei inputs due to significant nuclei loss during HCR wash steps, and scaled-up reactions may be necessary to recover sufficient nuclei.

Our oligodendrocyte study only analyzed two ages (2 and 25 months) representing the extreme ends of the murine lifespan; consequently, some observations may reflect developmental rather than aging-related changes. Including intermediate ages would help clarify these changes. Finally, while we validated *Zbtb16* exon 7 and mapped other DEEs to functional domains, further work is needed to confirm the biological relevance of individual exon-level findings.

RESOURCE AVAILABILITY

Lead contact

Requests for further information should be directed to the lead contact, Junyue Cao (jcao@rockefeller.edu).

Materials availability

This study did not generate new unique reagents.

Data and code availability

Raw and processed data are available from GEO (GEO: GSE295135). Processed Monocle CellDataSet objects can be downloaded from Zenodo: https://doi.org/10.5281/zenodo.15393823. The EasySci computational pipeline for processing EnrichSci data is available on Zenodo: https://doi.org/10.5281/zenodo.8395492. Computational notebooks for analyses in this study are available at Github: https://github.com/andrewliao99/EnrichSci_analysis (https://doi.org/10.5281/zenodo.17497563).

ACKNOWLEDGMENTS

We thank all members of the Cao Lab for helpful discussions and feedback. We also thank members of the Rockefeller University Information Technology and High-Performance Computing team, especially J. Banfelder and B. Jayaraman, for their great support. This research was supported by the G. Harold and Leila Y. Mathers Charitable Foundation as well as the Stavros Niarchos Foundation (SNF) as part of its grant to the SNF Institute for Global Infectious Disease Research at the Rockefeller University. This work was funded by NIH grants (1DP2HG012522, 1R01AG076932, and RM1HG011014) and the Mathers Foundation to J.C. This work was also supported by the Hevolution Foundation/American Federation of Aging Research New Investigator Awards in Aging Biology and Geroscience Research to J.C. W.Z. was funded by the Kellen Women's Entrepreneurship Fund and the Black Family Therapeutic Development Fund. A.L. was supported by a Medical Scientist Training Program grant from the National Institute of General Medical Sciences of the NIH (T32GM152349) to the Weill Cornell/Rockefeller/Sloan Kettering Tri-Institutional MD-PhD Program. This research was also supported in part by the Intramural Research Program of the NIH, grants ZIA AG000696 and ZIA AG000493, to M.M. The contributions of the NIH authors are considered works of the US government. The findings and conclusions presented in this paper are those of the authors and do not necessarily reflect the views of the NIH or the US Department of Health and Human Services.

AUTHOR CONTRIBUTIONS

J.C. and W.Z. conceptualized and supervised the project. A.L. performed all experiments, including technique development and optimization and computational analyses, with input from the other co-authors. J.C., W.Z., and A.L. wrote the manuscript with input and biological insight from all co-authors.

DECLARATION OF INTERESTS

The authors declare no competing interests.

DECLARATION OF GENERATIVE AI AND AI-ASSISTED TECHNOLOGIES IN THE WRITING PROCESS

While preparing this manuscript, the authors used ChatGPT to improve clarity and refine phrasing. All generated content was subsequently reviewed and edited by the authors, who take full responsibility for the final text.

STAR★METHODS

Detailed methods are provided in the online version of this paper and include the following:

- KEY RESOURCES TABLE
- EXPERIMENTAL MODEL AND STUDY PARTICIPANT DETAILS
  - Cell culture
  - Animals
- METHOD DETAILS
  - Cell line harvesting and nuclei isolation
  - Mouse brain collection and nuclei isolation
  - EnrichSci HCR protocol
  - HCR oligonucleotide probe generation
  - Cell line mixture HCR
  - Oligodendrocyte-targeted HCR
  - FACS enrichment of target cell populations
  - HCR nuclei input numbers and reaction scaling
  - EnrichSci snRNA-seq protocol
  - Reverse transcripion (RT)
  - Pooling, washing, and ligation

CellPress
OPEN ACCESS

- Pooling, washing, and second strand synthesis (SSS)
- 1× AMPure beads purification and tagmentation
- SDS treatment and PCR
- Library purification and sequencing
- EnrichSci data processing
- Cell filtering, clustering, and annotation for EnrichSci
- Cell population dynamics analysis
- Differential expression analysis
- Gene set enrichment analysis
- Exon–domain mapping analysis
- Splicing factor target enrichment analysis

SUPPLEMENTAL INFORMATION

Supplemental information can be found online at https://doi.org/10.1016/j.xgen.2025.101101.

Received: May 20, 2025
Revised: September 29, 2025
Accepted: November 11, 2025
Published: December 11, 2025

REFERENCES

1. Sziraki, A., Lu, Z., Lee, J., Banyai, G., Anderson, S., Abdulraouf, A., Metzner, E., Liao, A., Banfelder, J., Epstein, A., et al. (2023). A global view of aging and Alzheimer's pathogenesis-associated cell population dynamics and molecular signatures in human and mouse brains. Nat. Genet. *55*, 2104–2116.
2. Zhang, Z., Schaefer, C., Jiang, W., Lu, Z., Lee, J., Sziraki, A., Abdulraouf, A., Wick, B., Haeussler, M., Li, Z., et al. (2025). A panoramic view of cell population dynamics in mammalian aging. Science *387*, eadn3949.
3. Amamoto, R., Garcia, M.D., West, E.R., Choi, J., Lapan, S.W., Lane, E.A., Perrimon, N., and Cepko, C.L. (2019). Probe-Seq enables transcriptional profiling of specific cell types from heterogeneous tissue by RNA-based isolation. eLife *8*, e51452.
4. Clark, I.C., Wheeler, M.A., Lee, H.G., Li, Z., Sanmarco, L.M., Thaploo, S., Polonio, C.M., Shin, S.W., Scalisi, G., Henry, A.R., et al. (2023). Identification of astrocyte regulators by nucleic acid cytometry. Nature *614*, 326–333.
5. Abay, T., Stickels, R.R., Takizawa, M.T., Nalbant, B.N., Hsieh, Y.H., Hwang, S., Snopkowski, C., Yu, K.K.H., Abou-Mrad, Z., Tabar, V., et al. (2025). Transcript-specific enrichment enables profiling of rare cell states via single-cell RNA sequencing. Nat. Genet. *57*, 451–460.
6. Cao, J., Packer, J.S., Ramani, V., Cusanovich, D.A., Huynh, C., Daza, R., Qiu, X., Lee, C., Furlan, S.N., Steemers, F.J., et al. (2017). Comprehensive single-cell transcriptional profiling of a multicellular organism. Science *357*, 661–667.
7. Choi, H.M.T., Schwarzkopf, M., Fornace, M.E., Acharya, A., Artavanis, G., Stegmaier, J., Cunha, A., and Pierce, N.A. (2018). Third-generation hybridization chain reaction: multiplexed, quantitative, sensitive, versatile, robust. Development *145*, dev165753.
8. Schwarzkopf, M., Liu, M.C., Schulte, S.J., Ives, R., Husain, N., Choi, H.M.T., and Pierce, N.A. (2021). Hybridization chain reaction enables a unified approach to multiplexed, quantitative, high-resolution immunohistochemistry and in situ hybridization. Development *148*, dev199847.
9. Rosenberg, A.B., Roco, C.M., Muscat, R.A., Kuchina, A., Sample, P., Yao, Z., Graybuck, L.T., Peeler, D.J., Mukherjee, S., Chen, W., et al. (2018). Single-cell profiling of the developing mouse brain and spinal cord with split-pool barcoding. Science *360*, 176–182.
10. Chomczynski, P. (1992). Solubilization in formamide protects RNA from degradation. Nucleic Acids Res. *20*, 3791–3792.
11. Lehrach, H., Diamond, D., Wozney, J.M., and Boedtker, H. (1977). RNA molecular weight determinations by gel electrophoresis under denaturing conditions, a critical reexamination. Biochemistry *16*, 4743–4751.
12. Takara, B. (2015). Extending the Boundaries of Reverse Transcription. https://www.takarabio.com/documents/Application%20Note/633429_PrimeScript_AN_0415_IN.pdf?srsltid=AfmBOopqi4bR9_9x3Ncqdm3MwJGcM-rbd3rHPHgzN-XUN8Kv-Lkzx_2Y.
13. Zhang, X., Huang, N., Xiao, L., Wang, F., and Li, T. (2021). Replenishing the Aged Brains: Targeting Oligodendrocytes and Myelination? Front. Aging Neurosci. *13*, 760200.
14. Yao, Z., van Velthoven, C.T.J., Kunst, M., Zhang, M., McMillen, D., Lee, C., Jung, W., Goldy, J., Abdelhak, A., Aitken, M., et al. (2023). A high-resolution transcriptomic and spatial atlas of cell types in the whole mouse brain. Nature *624*, 317–332.
15. Hao, Y., Hao, S., Andersen-Nissen, E., Mauck, W.M., III, Zheng, S., Butler, A., Lee, M.J., Wilk, A.J., Darby, C., Zager, M., et al. (2021). Integrated analysis of multimodal single-cell data. Cell *184*, 3573–3587.e29.
16. Pandey, S., Shen, K., Lee, S.H., Shen, Y.A.A., Wang, Y., Otero-García, M., Kotova, N., Vito, S.T., Laufer, B.I., Newton, D.F., et al. (2022). Disease-associated oligodendrocyte responses across neurodegenerative diseases. Cell Rep. *40*, 111189.
17. Chamling, X., Kallman, A., Fang, W., Berlinicke, C.A., Mertz, J.L., Devkota, P., Pantoja, I.E.M., Smith, M.D., Ji, Z., Chang, C., et al. (2021). Single-cell transcriptomic reveals molecular diversity and developmental heterogeneity of human stem cell-derived oligodendrocyte lineage cells. Nat. Commun. *12*, 652.
18. Jin, K., Yao, Z., van Velthoven, C.T.J., Kaplan, E.S., Glattfelder, K., Barlow, S.T., Boyer, G., Carey, D., Casper, T., Chakka, A.B., et al. (2025). Brain-wide cell-type-specific transcriptomic signatures of healthy ageing in mice. Nature *638*, 182–196.
19. Lu, Z., Zhang, M., Lee, J., Sziraki, A., Anderson, S., Zhang, Z., Xu, Z., Jiang, W., Ge, S., Nelson, P.T., et al. (2023). Tracking cell-type-specific temporal dynamics in human and mouse brains. Cell *186*, 4345–4364.e24.
20. Dann, E., Henderson, N.C., Teichmann, S.A., Morgan, M.D., and Marioni, J.C. (2022). Differential abundance testing on single-cell data using k-nearest neighbor graphs. Nat. Biotechnol. *40*, 245–253.
21. Ximerakis, M., Lipnick, S.L., Innes, B.T., Simmons, S.K., Adiconis, X., Dionne, D., Mayweather, B.A., Nguyen, L., Niziolek, Z., Ozek, C., et al. (2019). Single-cell transcriptomic profiling of the aging mouse brain. Nat. Neurosci. *22*, 1696–1708.
22. Allen, W.E., Blosser, T.R., Sullivan, Z.A., Dulac, C., and Zhuang, X. (2023). Molecular and spatial signatures of mouse brain aging at single-cell resolution. Cell *186*, 194–208.e18.
23. Qiu, X., Hill, A., Packer, J., Lin, D., Ma, Y.A., and Trapnell, C. (2017). Single-cell mRNA quantification and differential analysis with Census. Nat. Methods *14*, 309–315.
24. Kolberg, L., Raudvere, U., Kuzmin, I., Adler, P., Vilo, J., and Peterson, H. (2023). g:Profiler-interoperable web service for functional enrichment analysis and gene identifier mapping (2023 update). Nucleic Acids Res. *51*, W207–W212.
25. Ashburner, M., Ball, C.A., Blake, J.A., Botstein, D., Butler, H., Cherry, J.M., Davis, A.P., Dolinski, K., Dwight, S.S., Eppig, J.T., et al. (2000). Gene ontology: tool for the unification of biology. The Gene Ontology Consortium. Nat. Genet. *25*, 25–29.
26. Gene Ontology Consortium; Aleksander, S.A., Balhoff, J., Carbon, S., Cherry, J.M., Drabkin, H.J., Ebert, D., Feuermann, M., Gaudet, P., Harris, N.L., et al. (2023). The Gene Ontology knowledgebase in 2023. Genetics *224*, iyad031.
27. Usui, N., Berto, S., Konishi, A., Kondo, M., Konopka, G., Matsuzaki, H., and Shimada, S. (2021). Zbtb16 regulates social cognitive behaviors and neocortical development. Transl. Psychiatry *11*, 242.
28. Rehman, A., Duffy, M., Gresova, K., Belair, C., Yang, N., Wang, L., Lee, C.T., Payea, M., Malla, S., Dar, S.A., et al. (2025). Cell-specific RNA

isoform remodeling in the aging mouse brain. Preprint at bioRxiv. https://doi.org/10.1101/2025.06.05.658133.

29. Nowicka, M., and Robinson, M.D. (2016). DRIMSeq: a Dirichlet-multinomial framework for multivariate count outcomes in genomics. F1000Res. *5*, 1356.
30. Paysan-Lafosse, T., Andreeva, A., Blum, M., Chuguransky, S.R., Grego, T., Pinto, B.L., Salazar, G.A., Bileschi, M.L., Llinares-López, F., Meng-Papaxanthos, L., et al. (2025). The Pfam protein families database: embracing AI/ML. Nucleic Acids Res. *53*, D523–D534.
31. Yang, Y.C.T., Di, C., Hu, B., Zhou, M., Liu, Y., Song, N., Li, Y., Umetsu, J., and Lu, Z.J. (2015). CLIPdb: a CLIP-seq database for protein-RNA interactions. BMC Genom. *16*, 51.
32. Huang, X., Henck, J., Qiu, C., Sreenivasan, V.K.A., Balachandran, S., Amarie, O.V., Hrabě de Angelis, M., Behncke, R.Y., Chan, W.L., Despang, A., et al. (2023). Single-cell, whole-embryo phenotyping of mammalian developmental disorders. Nature *623*, 772–781.
33. Gabitto, M.I., Travaglini, K.J., Rachleff, V.M., Kaplan, E.S., Long, B., Ariza, J., Ding, Y., Mahoney, J.T., Dee, N., Goldy, J., et al. (2024). Integrated multimodal cell atlas of Alzheimer's disease. Nat. Neurosci. *27*, 2366–2383.
34. Cao, J., Zhou, W., Steemers, F., Trapnell, C., and Shendure, J. (2020). Sci-fate characterizes the dynamics of gene expression in single cells. Nat. Biotechnol. *38*, 980–988.
35. Xu, Z., Sziraki, A., Lee, J., Zhou, W., and Cao, J. (2024). Dissecting key regulators of transcriptome kinetics through scalable single-cell RNA profiling of pooled CRISPR screens. Nat. Biotechnol. *42*, 1218–1223.
36. Suliman, B.A., Xu, D., and Williams, B.R.G. (2012). The promyelocytic leukemia zinc finger protein: two decades of molecular oncology. Front. Oncol. *2*, 74.
37. Liu, T.M., Lee, E.H., Lim, B., and Shyh-Chang, N. (2016). Concise Review: Balancing Stem Cell Self-Renewal and Differentiation with PLZF. Stem Cell. *34*, 277–287.
38. Lee, S., Kim, T.K., Choi, J.E., Kim, H.-S., and Im, H.-I. (2022). Striatal ZBTB16 Is Associated With Cognitive Deficits in Alzheimer Disease Mice. Int. Neurourol. J. *26*, S106–S116.
39. Dong, D., Du, Y., Fei, X., Yang, H., Li, X., Yang, X., Ma, J., Huang, S., Ma, Z., Zheng, J., et al. (2023). Inflammasome activity is controlled by ZBTB16-dependent SUMOylation of ASC. Nat. Commun. *14*, 8465.
40. Alfonso-Gonzalez, C., and Hilgers, V. (2024). Alternative) transcription start sites as regulators of RNA processing. Trends Cell Biol. *34*, 1018–1028.
41. Ren, F., Zhang, N., Zhang, L., Miller, E., and Pu, J.J. (2020). Alternative Polyadenylation: a new frontier in post transcriptional regulation. Biomark. Res. *8*, 67.
42. Bedi, K., Magnuson, B., Narayanan, I.V., McShane, A., Ashaka, M., Paulsen, M.T., Wilson, T.E., and Ljungman, M. (2024). Isoform and pathway-specific regulation of post-transcriptional RNA processing in human cells. Preprint at bioRxiv. https://doi.org/10.1101/2024.06.12.598705.
43. Cao, J., Cusanovich, D.A., Ramani, V., Aghamirzaie, D., Pliner, H.A., Hill, A.J., Daza, R.M., McFaline-Figueroa, J.L., Packer, J.S., Christiansen, L., et al. (2018). Joint profiling of chromatin accessibility and gene expression in thousands of single cells. Science *361*, 1380–1385.
44. Mulqueen, R.M., Pokholok, D., Norberg, S.J., Torkenczy, K.A., Fields, A.J., Sun, D., Sinnamon, J.R., Shendure, J., Trapnell, C., O'Roak, B.J., et al. (2018). Highly scalable generation of DNA methylation profiles in single cells. Nat. Biotechnol. *36*, 428–431.
45. Cao, J., Spielmann, M., Qiu, X., Huang, X., Ibrahim, D.M., Hill, A.J., Zhang, F., Mundlos, S., Christiansen, L., Steemers, F.J., Trapnell, C., and Shendure, J. (2019). The single-cell transcriptional landscape of mammalian organogenesis. Nature *566*, 496–502.
46. Li, H., Handsaker, B., Wysoker, A., Fennell, T., Ruan, J., Homer, N., Marth, G., Abecasis, G., and Durbin, R. (2009). 1000 Genome Project Data Processing Subgroup. The Sequence Alignment/Map format and SAMtools. Bioinformatics *25*, 2078–2079.
47. Wolf, F.A., Angerer, P., and Theis, F.J. (2018). SCANPY: large-scale single-cell gene expression data analysis. Genome Biol. *19*, 15.
48. Wolock, S.L., Lopez, R., and Klein, A.M. (2019). Scrublet: Computational Identification of Cell Doublets in Single-Cell Transcriptomic Data. Cell Syst. *8*, 281–291.e9.
49. Dobin, A., Davis, C.A., Schlesinger, F., Drenkow, J., Zaleski, C., Jha, S., Batut, P., Chaisson, M., and Gingeras, T.R. (2013). STAR: ultrafast universal RNA-seq aligner. Bioinformatics *29*, 15–21.
50. Kuehn, E., Clausen, D.S., Null, R.W., Metzger, B.M., Willis, A.D., and Özpolat, B.D. (2022). Segment number threshold determines juvenile onset of germline cluster expansion in Platynereis dumerilii. J. Exp. Zool. B Mol. Dev. Evol. *338*, 225–240.
51. Perez, G., Barber, G.P., Benet-Pages, A., Casper, J., Clawson, H., Diekhans, M., Fischer, C., Gonzalez, J.N., Hinrichs, A.S., Lee, C.M., et al. (2025). The UCSC Genome Browser database: 2025 update. Nucleic Acids Res. *53*, D1243–D1249.

CellPress
OPEN ACCESS

STAR★METHODS

KEY RESOURCES TABLE

REAGENT or RESOURCE	SOURCE	IDENTIFIER
Biological samples		
Whole mouse brains	This study	N/A
Chemicals, peptides, and recombinant proteins		
AMPure XP Beads for DNA Cleanup	Beckman Coulter	Cat#A63882
BSA	NEB	Cat#B90000S
DAPI	Invitrogen	Cat#D1306
DMEM, high glucose	Gibco	Cat#11965118
dNTP Mix (10 mM each)	Thermo Scientific Chemicals	Cat#R0194
Fetal Bovine Serum	Sigma-Aldrich	Cat#F4135
Formaldehyde, 4% in PBS	Thermo Scientific Chemicals	Cat#J60401.AK
HCR (v3.0) Amplification Buffer	Molecular Instruments	N/A
HCR (v3.0) Amplifiers (B1-647)	Molecular Instruments	N/A
HCR (v3.0) Probe Hybridization Buffer	Molecular Instruments	N/A
HCR (v3.0) Wash Buffer	Molecular Instruments	N/A
Maxima H Minus Reverse Transcriptase (200 U/μL)	Thermo Scientific	Cat#EP0752
NEBNext High-Fidelity 2× PCR Master Mix	NEB	Cat#M0541L
NEBNext Ultra II Non-Directional RNA Second Strand Synthesis Module	NEB	Cat#E6111L
Penicillin-Streptomycin	Gibco	Cat#15140122
SUPERase·In™ RNase Inhibitor	Invitrogen	Cat#AM2696
T4 DNA Ligase (400,000 units/mL)	NEB	Cat#M0202LVIAL
T4 DNA Ligase Reaction Buffer	NEB	Cat#B0202SVIAL
TWEEN 20	Sigma-Aldrich	Cat#P9416-50ML
Critical commercial assays		
HCR RNA FISH (v3.0) Kit for Cells in Suspension	Molecular Instruments	N/A
Deposited data		
Raw and analyzed data	This study	GEO: GSE295135
Single-nucleus RNA-seq data of whole mouse brain across three age groups and two Alzheimer's disease associated mutants	Sziraki et al.[1]	GEO: GSE212606
Long-read single-cell RNA-seq data of mouse brains across the whole murine lifespan	Rehman et al.[28]	https://doi.org/10.1101/2025.06.05.658133
MERFISH data of young and aged mouse brains	Allen et al.[22]	https://cellxgene.cziscience.com/collections/31937775-0602-4e52-a799-b6acdd2bac2e
Experimental models: Cell lines		
A375	ATCC	N/A
HeLa	ATCC	N/A
HEK293T	ATCC	N/A
NIH/3T3	ATCC	N/A
Experimental models: Organisms/strains		
Mouse: C57BL/6J	The Jackson Laboratory	RRID:IMSR_JAX:000664
Oligonucleotides		
See Table S1	IDT	N/A

(Continued on next page)

Continued

REAGENT or RESOURCE	SOURCE	IDENTIFIER
Software and algorithms		
bcl2fastq	Illumina	https://support.illumina.com/sequencing/sequencing_software/bcl2fastq-conversion-software.html
CLIPdb	Yang et al.[31]	http://clipdb.ncrnalab.org
g:profiler	Kolberg et al.[24]	https://biit.cs.ut.ee/gprofiler.
miloR	Dann et al.[20]	https://github.com/MarioniLab/miloR
Monocle 2	Qiu et al.[23]	https://github.com/cole-trapnell-lab/monocle2-rge-paper
Monocle 3	Cao et al.[45]	https://github.com/cole-trapnell-lab/monocle3
Python	Python Software Foundation	https://www.python.org/
R	R Core	https://www.r-project.org/
Samtools	Li et al.[46]	http://www.htslib.org/download/
Scanpy	Wolf et al.[47]	https://github.com/scverse/scanpy
Scrublet	Wolock et al.[48]	https://github.com/swolock/scrublet
Seurat	Hao et al.[15]	https://satijalab.org/seurat/
STAR	Dobin et al.[49]	https://github.com/alexdobin/STAR
Trim Galore	Babraham Institute	https://github.com/FelixKrueger/TrimGalore

EXPERIMENTAL MODEL AND STUDY PARTICIPANT DETAILS

Cell culture

HEK293T, A375, HeLa, and NIH/3T3 cell lines were cultured in 10 cm dishes at 37°C with 5% CO_2 in high glucose DMEM (Gibco, 11965-118) supplemented with 10% Fetal Bovine Serum (Sigma-Aldrich, F4135) and 1% penicillin-streptomycin (Gibco, 15140-122). All cell lines were obtained from the American Type Culture Collection. Cell line authentication was not performed, but all lines exhibited expected growth characteristics and marker gene expression.

Animals

C57BL/6 wild-type mice at 2 months (n = 3) and 25 months (n = 3) were obtained from The Jackson Laboratory. Mice were housed socially and maintained on a regular 12h/12h day/night cycle. Euthanization and tissue collection were performed on the same day within a 90-min window to control for circadian effects. Mice were euthanized using inhalation of carbon dioxide (CO_2), followed by cervical dislocation, prior to tissue dissection. All animal procedures were in accordance with institutional, state, and government regulations and approved under the IACUC protocol 24012-H.

METHOD DETAILS

Cell line harvesting and nuclei isolation

At 80–90% confluency in a 10 cm dish, cell lines were harvested with 0.25% trypsin-EDTA, washed with PBS, and lysed in 2 mL of hypotonic lysis buffer with sucrose (7.68 mM Na2HPO4·2H2O, 4.49 mM $NaH_2PO_4{\cdot}H_2O$, 1.76 mM KH_2PO_4, 2.68 mM KCl, 10.27 mM NaCl, 3 mM MgCl2, 0.33 M sucrose, 0.025% IEGPAL, 1% DEPC). Lysis was performed in 5 mL tubes incubated on a rotator at 4°C for 20 min. After lysis, nuclei were pelleted for 5 min at 500g (4°C), resuspended in 2 mL of 0.4% formaldehyde in PBST, and fixed for 15 min on a rotator at 4°C. Nuclei were then pelleted for 5 min at 1,200g (4°C) and washed once with 2 mL of Nuclei Suspension Buffer (NSB) (10 mM Tris-HCl pH 7.5 (VWR, 97062-936), 10 mM NaCl (VWR, 97062-858), 3 mM MgCl2 (VWR, 97062-848), supplemented with 0.1% SUPERase·In RNase Inhibitor (Thermo Fisher Scientific, AM2696), 1% BSA (NEB, B9200S), and 0.1% Tween 20 (Sigma, P9416-100ML). After resuspension in NSB, nuclei were counted and used directly in the HCR protocol. For long-term storage, nuclei were diluted to ~10 million nuclei/mL in NSB with 10% DMSO, cryopreserved at −80°C using a controlled-rate freezing container (Corning, 07-210-009), and kept at −80°C until usage.

Mouse brain collection and nuclei isolation

After euthanization, whole brains were extracted from mice, immediately snap-frozen in liquid nitrogen, and stored at −80°C until usage. To isolate nuclei, thawed brains were first diced into fine pieces (<1 mm^3) using carbon steel razor blades (VWR, 100491-872) in a 6 cm dish containing 1 mL hypotonic lysis buffer with sucrose. All contents of the dish were transferred to a 15 mL tube

containing 14 mL of hypotonic lysis buffer with sucrose for a 25 min incubation while rotating at 4°C, and then homogenized through 40 μm cell strainers (Ward's Science, 470236-276). Extracted nuclei were then pelleted, fixed in 10 mL 0.4% formaldehyde in PBST while rotating for 15 min at 4°C, and washed with 10 mL of NSB. Nuclei were counted and resuspended at ~10 million nuclei/mL in NSB with 10% DMSO, cryopreserved at −80°C using a controlled-rate freezing container, and stored at −80°C until usage. After extraction and fixation, nuclei yields ranged from ~65 million in young brains to ~110 million in larger aged brains.

EnrichSci HCR protocol

HCR was performed largely according to the Molecular Instruments (MI) "*HCR RNA flow cytometry protocol for mammalian cells in suspension*" protocol (version dated 2023-02-13), with specific optimizations made for compatibility with nuclei, improved nuclei recovery, and optimal signal-to-noise. Of note, extracted nuclei were fixed with 0.4% formaldehyde, in contrast to 4% formaldehyde fixation and ethanol permeabilization for whole cells in the MI protocol, and stored at −80°C until usage. We have observed a slight reduction in HCR signal using frozen versus freshly extracted nuclei, although overall signal-to-noise is well-maintained. Decreased HCR efficiency was also noted with increased freeze–thaw cycles of HCR reagents; therefore, we recommend aliquoting all reagents for long-term storage. To minimize nuclei loss during the HCR workflow, the number of wash steps during the detection and amplification stages was significantly reduced by 7 washes from the original MI protocol. All centrifugation steps to pellet nuclei were performed at 1,200g (4°C) for 5 min unless otherwise specified. All incubations were performed using a rotating mixer (Miltenyi Biotec, 130-090-753). Finally, to ensure recovery of sufficient numbers of nuclei in the oligodendrocyte profiling experiment, we scaled up the probe hybridization reaction volumes from 1× as described in the MI protocol to 5×. At the pre-amplification step, the reaction was scaled back to the 1× reaction volume in the MI protocol.

HCR oligonucleotide probe generation

HCR probes used in this study were designed using the custom probe generator[50] developed by the Özpolat Lab (available at: https://github.com/rwnull/insitu_probe_generator). In brief, the mRNA sequence for each target gene was retrieved from the UCSC Genome Browser[51] and provided as input to the pipeline. Standard parameters were used for most targets, including skipping the first 100 bp of the transcript (when transcript length permitted) and limiting homopolymer runs of polyA/T and polyC/G to a maximum of 5 and 4 bp, respectively. Candidate probes were then BLASTed against the mouse genome (mm39), and any probes with predicted off-target binding were removed by the pipeline. From the resulting list of probe pairs, we manually selected 5 to 11 pairs per gene, prioritizing probes with GC content between 45 and 60% and within 6% of one another and ensuring spacing across the transcript to maximize coverage. Individual oligonucleotide probes were ordered from Integrated DNA Technologies (IDT) in 96-well plates at 200 μM in IDTE and pooled into probe sets for downstream use.

Cell line mixture HCR

Nuclei from NIH/3T3, HEK293T, A375, and HeLa cells were extracted and fixed as described above, and HCR was performed directly without nuclei freezing for the cell line mixture experiments. To generate the cell line mixture, 250,000 fixed nuclei from each cell line (or 1 million nuclei for individual controls) were combined in a 1.5 mL tube, pelleted, and resuspended in 400 μL of MI probe hybridization buffer pre-warmed to 37°C. After a 30-min pre-hybridization incubation at 37°C, 100 μL of probe solution (prepared by adding 2 μL of probe stock at 2 μM per probe to 96 μL of hybridization buffer) was added to each sample to achieve a final concentration of 8 nM per probe, and samples were incubated overnight at 37°C.

The following day, 850 μL of MI probe wash buffer, pre-warmed to 37°C, was added to each sample before pelleting at 1,700g (4°C) for 5 min. Supernatant was removed, and pellets were resuspended in 1 mL of probe wash buffer and incubated at 37°C for 10 min. Each sample was then pelleted at 1,600g (4°C) for 5 min, resuspended in 1 mL of 5× SSCT (5× saline sodium citrate, 0.1% Tween 20), and incubated at room temperature for 5 min. Samples were pelleted, resuspended in 150 μL of MI amplification buffer, and incubated at room temperature for 30 min in a pre-amplification step. After pre-amplification, 110 μL of amplifier solution (prepared by adding 5 μL each of MI amplifier h1 and h2 to 100 μL of amplification buffer) was added to each sample. Samples were incubated in the dark overnight at room temperature.

The following day, 1 mL of 5× SSCT was added to each sample before pelleting at 1,500g (4°C) for 5 min. Samples were then washed in 1 mL of 5× SSCT, resuspended in 400 μL NSB containing DAPI (Thermo Fisher Scientific, D1306) at a 1:100 dilution from a 0.25 mg/mL stock, and sonicated for 8 s at low power (Diagenode, B01020014) before proceeding to FACS.

Oligodendrocyte-targeted HCR

Nuclei from young and aged mouse brains were extracted, fixed, and frozen as described above. 5 million frozen, fixed nuclei from each sample were thawed from −80°C in a 37°C water bath, pelleted, and resuspended in 2 mL of MI probe hybridization buffer pre-warmed to 37°C. After a 30-min pre-hybridization incubation at 37°C, 500 μL of probe solution (prepared by adding 5 μL of probe stock at 2 μM per probe to 495 μL of hybridization buffer) was added to each sample to achieve a final concentration of 4 nM per probe, and samples were incubated overnight at 37°C.

The following day, 2.5 mL of MI probe wash buffer, pre-warmed to 37°C, was added to each sample before pelleting at 1,700g (4°C) for 5 min. Supernatant was removed, and pellets were resuspended in 5 mL of probe wash buffer and incubated at 37°C for 10 min. Each sample was then pelleted at 1,600g (4°C) for 5 min, resuspended in 5 mL of 5× SSCT (5× saline sodium citrate, 0.1% Tween 20),

and incubated at room temperature for 5 min. Samples were pelleted, resuspended in 150 μL of MI amplification buffer, transferred to a 1.5 mL tube, and incubated at room temperature for 30 min in a pre-amplification step. After pre-amplification, 110 μL of amplifier solution (prepared by adding 5 μL each of MI amplifier h1 and h2 to 100 μL of amplification buffer) was added to each sample. Samples were incubated in the dark overnight at room temperature.

The following day, 1 mL of 5× SSCT was added to each sample before pelleting at 1,500g (4°C) for 5 min. Samples were then washed in 1 mL of 5× SSCT, resuspended in 400 μL NSB containing DAPI at a 1:100 dilution from a 0.25 mg/mL stock, and sonicated for 8 s at low power before proceeding to FACS.

FACS enrichment of target cell populations

FACS was performed using an SH800 Cell Sorter with a 100 μM sorting chip (Sony, #LE-C3210). Nuclei were first gated to select DAPI-positive singlets, followed by gating for populations of interest based on HCR fluorescent signal. Sorting was carried out into 1.5 mL tubes pre-coated with NSB. To coat tubes, 1 mL of NSB was added, vortexed, and removed, followed by brief centrifugation to collect and aspirate residual buffer. Minimizing residual buffer was critical to ensure the intended nuclei concentration in the sorted solution. The 100 μM chip sorts events in 3 nL droplets to yield a sorted solution concentrated at approximately 333 nuclei/μL. After sorting, tubes were pelleted to collect all solution from the walls, but the supernatant was not removed. Instead, the pelleted nuclei were gently resuspended in the solution, which was directly used at the sorted concentration for reverse transcription.

HCR nuclei input numbers and reaction scaling

To account for nuclei loss during the HCR protocol as well as the downstream snRNA-seq library preparation steps, nuclei input numbers were scaled accordingly. For cell lines, we recovered ~30% (300,000 nuclei) of the initial input number (1 million nuclei) as DAPI singlets during sorting. For mouse brain nuclei, singlet recovery was lower at ~10% of the input. Thus, we scaled up the oligodendrocyte-targeting reaction volumes and inputs by 5× (5 million nuclei per sample), yielding ~500,000 nuclei singlets during sorting. Roughly 2.6% (~10,000 nuclei) of singlets were gated as oligodendrocytes and used as input for reverse transcription. After additional nuclei loss during library preparation and filtering of low-quality nuclei during data processing, ~30% of the sorted input was ultimately recovered, ranging from 2,419 to 3,472 high-quality nuclei per sample.

EnrichSci snRNA-seq protocol

Following HCR and FACS, sorted nuclei underwent combinatorial indexing-based sequencing library generation largely according to the EasySci[1] protocol. For all major experiments described in this study, reverse transcription through second strand synthesis was performed immediately after FACS. However, as noted in the PERFF-seq protocol,[5] we have found that samples stored at 4°C for up to 6 days post-FACS also produced usable data for test experiments, demonstrating robustness of the workflow to delayed processing (Figures S1G–S1I).

Reverse transcripion (RT)

After measuring the volume of sorted nuclei solution, 1 μL of 10 mM dNTP was added into the same tube for every 8 μL of sorted nuclei solution. After gently mixing, 2.26 μL of nuclei + dNTP solution (~666 nuclei) was added to each RT well in a 96-well plate on ice (Genesee Scientific, #24–302). 0.5 μL of 50 μM well-specific oligo-dT primers and 0.5 μL of 50 μM well-specific random hexamer primers were then added to provide the first round of indexing. The plate was incubated at 55°C for 5 min then placed back on ice. Next, 1.75 μL of RT master mix (for one plate, mix 110 μL of 5× Maxima RT buffer, 27.5 μL of Maxima H Minus Reverse Transcriptase, and 27.5 μL of SUPERase-In RNase Inhibitor, and 27.5 μL of nuclease-free water) was added to each well. The plate was then incubated on a temperature gradient (4°C, 10°C, 20°C, 30°C, 40°C, and 50°C for 2 min each, followed by 55°C for 15 min) before being placed back on ice.

Pooling, washing, and ligation

Following RT, 5 μL of NSB was added to each well, and all wells were pooled into a 1.5 mL tube. Pooled nuclei were pelleted and washed once with 1 mL of NSB, then resuspended in 260 μL of NSB. 2.5 μL of resuspended nuclei was added to each well in a new 96-well plate. 1 μL of 3.125 μM well-specific DNA ligation primer/adaptor complex was added each well. Finally, 1.5 μL of ligation master mix (for one plate, mix 55 μL of 10× T4 Ligation Buffer, 5.5 μL of SUPERase-In RNase Inhibitor, 55 μL of T4 DNA Ligase, and 47.5 μL of nuclease-free water) was added to each well. The plate was then incubated at room temperature for 30 min on a Fisherbrand Nutating Mixer (Fisher Scientific, #88-861-043) at 50 rpm before being placed back on ice.

Pooling, washing, and second strand synthesis (SSS)

Following ligation, 1 μL of 18 mM EDTA was added to each well, and all wells were pooled into a 1.5 mL tube. Pooled nuclei were pelleted and washed once with 1 mL of NSB, then resuspended to ~250 nuclei/μL (the total nuclei recovered at this step should be roughly half of the total nuclei input for RT). 4 μL of resuspended nuclei were distributed into each well of 4 PCR strips (more strips

or a plate can be used here if necessary). 1 μL of SSS master mix (for each well, mix 0.33 μL of NEBNext Ultra II Non-directional RNA SSS Enzyme Mix with 0.67 μL of SSS Reaction Buffer) was added to each well, and strips were incubated at 16°C for one hour. After SSS, samples were stored at −20°C overnight.

1× AMPure beads purification and tagmentation

Following SSS, 5 μL of DNA binding buffer was added to each well, mixed, and left to incubate for 5 min at room temperature. 10 μL of AMPure XP beads were then added to each well, mixed, and left to incubate for 5 min at room temperature. The strips were then placed on a magnetic rack for 5 min. The resulting supernatant was removed, and the beads were washed twice with 50 μL of freshly made 80% ethanol, briefly centrifuging and removing all residual ethanol after the second wash. Off the magnetic rack, 7 μL of elution buffer was added to each well, mixed, and left to incubate for 3 min at room temperature. The strips were then placed back on the magnetic rack and left to incubate for 3 min. Finally, 6.6 μL of solution was aspirated and transferred to new PCR strips. 6.6 μL of 1:100 Tagmentase:Tagmentation buffer mix was added to each well, and strips were incubated at 55°C for 5 min.

SDS treatment and PCR

Following tagmentation, 2.8 μL of SDS/P5 primer master mix (for each well, mix 0.4 μL SDS, 0.4 μL of BSA, and 2 μL of universal P5 primer) was added to each well, and strips were incubated at 55°C for 15 min before being placed back on ice. To each well, 2 μL of 10% Tween 20 was added, followed by 2 μL of indexed P7 primer. Finally, 20 μL of NEBNext High-Fidelity 2× PCR Master Mix was added to each well. Strips were incubated at 72°C for 5 min, 98°C for 30 s, and 15 cycles of 98°C for 10 s, 66°C for 30 s, and 72°C for 30 s, followed by a final extension at 72°C for 5 min. After the reaction, strips were placed back on ice.

Library purification and sequencing

All wells were pooled together following PCR, and 200 μL of pooled PCR product was used for a 0.8× AMPure beads purification. In a 1.5 mL tube, 160 μL of beads was added to 200 μL of pooled PCR product, mixed, and left to incubate at room temperature for 5 min. The tube was placed on a magnetic rack for 5 min, and the resulting supernatant was removed. The beads were washed twice with freshly made 80% ethanol, briefly centrifuging and removing all residual ethanol after the second wash. Off the magnetic rack, 105 μL of elution buffer was added, mixed, and left to incubate for 3 min at room temperature. The tube was placed back on the magnetic rack for 3 min, and 100 μL of solution was aspirated and transferred to a new tube. A final 0.9× AMPure beads purification was performed, eluting in a final volume of 20 μL. All libraries were visualized by gel electrophoresis, and concentrations were determined using a Qubit fluorometer (Invitrogen, Q33231). All libraries were sequenced on the NextSeq 1000 platform (Illumina) using a 100-cycle kit (Read 1: 58 cycles, Read 2: 60 cycles, Index 1: 10 cycles, Index 2: 10 cycles). The cell line library was sequenced to ∼13,000 reads per cell, and the mouse brain library was sequenced to ∼37,000 reads/cell.

EnrichSci data processing

Raw sequencing data were processed using the previously developed EasySci1 pipeline for read alignment and generation of gene and exon count matrices for snRNA-seq libraries. In brief, base calls were converted to FASTQ format and demultiplexed using Illumina bcl2fastq (v2.19.0.316), allowing up to one mismatch in barcode sequences (edit distance<2). RT barcodes were corrected to their nearest valid barcode (edit distance<2), and reads with barcodes that could not be corrected (edit distance≥2) were excluded. Adaptors and barcodes were trimmed, and Trim Galore (v0.4.1) was used to additionally remove poly(A) sequences and low-quality base calls. Using STAR[49] (v2.5.2b), trimmed reads were aligned to a chimeric human and mouse genome (hg27/mm10) for the cell line mixture experiment and the mouse genome (mm39) for mouse brain profiling. After removal of PCR duplicates, which share a unique molecular identifier (UMI) sequence, RT barcode, and tagmentation site, reads are split into SAM files per cell. A custom EasySci[1] script was used to quantify gene and exon expression per cell. To assign reads to genes, a read was counted if its aligned coordinates overlapped with annotated gene regions. If a read derived from the oligo-dT RT primer was ambiguous between multiple genes, it was assigned to the gene with the closest 3′ end. If a read did not initially map to a gene, the script also searched for potential gene assignments up to 1,000 bp upstream or on the opposite strand. After these steps, reads without assigned genes were discarded. A similar approach was performed to generate the exon count matrix.

Cell filtering, clustering, and annotation for EnrichSci

Gene and exon expression matrices were constructed from the raw sequencing data as described above. In the cell line mixture experiment, cells with less than 1000 UMIs and 100 unique genes were discarded. For mouse brain profiling, cells with less than 500 UMIs and 100 unique genes were discarded.

To identify doublets, Scrublet[48] (v0.2.3) with Scanpy[47] (v1.6.0) was applied to each gene count matrix using the following parameters: min_count = 3, min_cells = 3, vscore_percentile = 85, n_pc = 30, expected_doublet_rate = 0.06, sim_doublet_ratio = 2, n_neighbors = 30. Cells with doublet scores over 0.2 were annotated as doublets and discarded, along with any cells from doublet-derived sub-clusters. Finally, cells that passed initial filtering but appeared to be doublets, based on clustering results and expression of markers from multiple cell types, were also removed.

Using Seurat[15] (v4.0.2), dimension reduction was performed on the data first by PCA using 30 components and then with UMAP before Louvain clustering. For the cell line mixture experiment, cluster identity was annotated using individual cell line spike-ins as a

reference. For mouse brain profiling, we performed two independent experimental batches. Although both batches were integrated to define cell-state identities, downstream gene-expression analyses were restricted to the second batch—comprising three biological replicates per age group versus only two aged replicates in the first batch. Initial cell type annotations were generated using the Allen Institute for Brain Science MapMyCells tool.[14] We then removed all cells that did not have the class_name '31 OPC-Oligo' to filter for only oligodendrocyte lineage cells. For higher resolution clustering of these cells, we used Seurat[15] to integrate oligodendrocyte lineage cells from the two independent experiment batches by regressing out the effect of experimental batch and age group. Following integration, we performed dimension reduction and clustering, revealing shared and divergent cell states between the two age groups. For downstream analyses, only the second batch with all three replicate samples per age group was used. Oligodendrocyte lineage subtypes were annotated based on the previous MapMyCells[14] annotations along with expression of cell type-specific markers (Figure 2D).

Cell population dynamics analysis

To assess the effects of aging on cell population dynamics within the oligodendrocyte lineage, we applied miloR[20] (v1.3.1), a single-cell differential abundance testing framework that utilizes k-nearest neighbor (KNN) graphs. We first constructed a KNN graph on the UMAP space using the buildGraph() function with k = 60. Cell neighborhoods were defined with makeNhoods(), and cell counts per sample within each neighborhood were computed using countCells(). Differential abundance testing was performed using testNhoods(), with significance assessed at a spatial FDR threshold of 0.05. To visualize differential abundance neighborhoods, we initially tried the plotNhoodGraphDA() function, but it is sensitive to local noise and does not easily show the density of specific conditions. To better visualize age-related cell population shifts, we first expanded each neighborhood by including all cells within a fixed radius of its UMAP coordinates. We then calculated the proportion of young and aged cells in each neighborhood, labeling them as *Young-enriched* or *Aged-enriched* if one age group comprised more than 70.5% of the cells. These expanded neighborhoods were more robust to local variability and better captured broader population shifts. Finally, to visualize the distribution of age group-enriched neighborhoods, we used ggplot2 to overlay density contours on a UMAP plot of the cell neighborhoods.

Differential expression analysis

To identify differentially expressed (DE) genes and exons between young and aged mice in each of the two mature oligodendrocyte subtypes, we employed the likelihood ratio test to identify genes and exons significantly associated with a specific population, using the differentialGeneTest() function in Monocle[23] (v2.28.0). The test was applied to a combined object containing both gene- and exon-level features, which improved statistical power and reduced the false discovery rate (FDR). Filtering first for DE genes, we used the following cutoffs: FDR<0.05, fold change>1.5, and counts per million (CPM) in the maximum condition>25. To filter for DE exons, we used the same FDR and fold change cutoffs but lowered the CPM threshold (in the maximum condition) to >10, accounting for the lower expression levels of individual exons. To classify DE exons as either DEG-derived or non-DEG-derived, we re-filtered genes using the DE exon CPM cutoff (>10) instead of the DE gene cutoff (>25). Using the same cutoffs as DE exon filtering thus allowed us to determine that under the same criteria, the parent genes of non-DEG-derived DE exons were only detectable at the exonic and not the gene level.

Gene set enrichment analysis

To identify enriched pathways associated with DE genes and exons, we used g:Profiler[24] (version e112_e.g.,59_p19_25aa4782) with Benjamini-Hochberg FDR<0.05 applied. DE genes and non-DEG-derived DE exons for each mature oligodendrocyte subtype and age group were separately analyzed. DE gene inputs consisted of unranked mouse gene symbols, and DE exon inputs used the corresponding parent gene symbols. We queried the following pathway databases: GO Biological Process (2024-10-7 release), KEGG pathways (2024-01-22 release), Reactome pathways (2025-02-03 release), and WikiPathways (2025-01-10 release). For visualization, we manually selected biologically relevant pathways for DE genes and exons in each age group and mature oligodendrocyte subtype and plotted them using custom ggplot2-based scripts.

Exon–domain mapping analysis

To assess whether DEEs were enriched in specific protein functional domains, we developed a custom pipeline that first mapped each DEE to the coding sequence(s) of its associated transcripts. Next, we converted the genomic coordinates of the mapped coding sequences to amino acid positions. Finally, we matched these positions to those of annotated Pfam domains[30] for the associated transcripts, thus identifying protein families and domains coded for by the majority of DEEs.

Splicing factor target enrichment analysis

To investigate whether dysregulation of splicing factors is linked to exon-level changes in their target transcripts, we performed enrichment analysis for targets of DE splicing factors among DEEs. We first filtered DEGs for known splicing factors using the Gene Ontology database[25,26] and then searched public CLIP-seq[31] or comparable data to identify transcripts bound by DE splicing factors. Enrichment of DEG splicing factor targets among DEE parent genes was assessed relative to non-DEE parent genes using Fisher's exact test, with significant enrichment indicating a potential link between age-dependent splicing factor dysregulation and exon-level expression dynamics.

Cell Genomics, Volume *6*

Supplemental information

Transcript-guided targeted cell enrichment

for scalable single-nucleus RNA sequencing

Andrew Liao, Zehao Zhang, Andras Sziraki, Abdulraouf Abdulraouf, Abid Rehman, Zihan Xu, Ziyu Lu, Weirong Jiang, Alia Arya, Jasper Lee, Manolis Maragkakis, Wei Zhou, and Junyue Cao

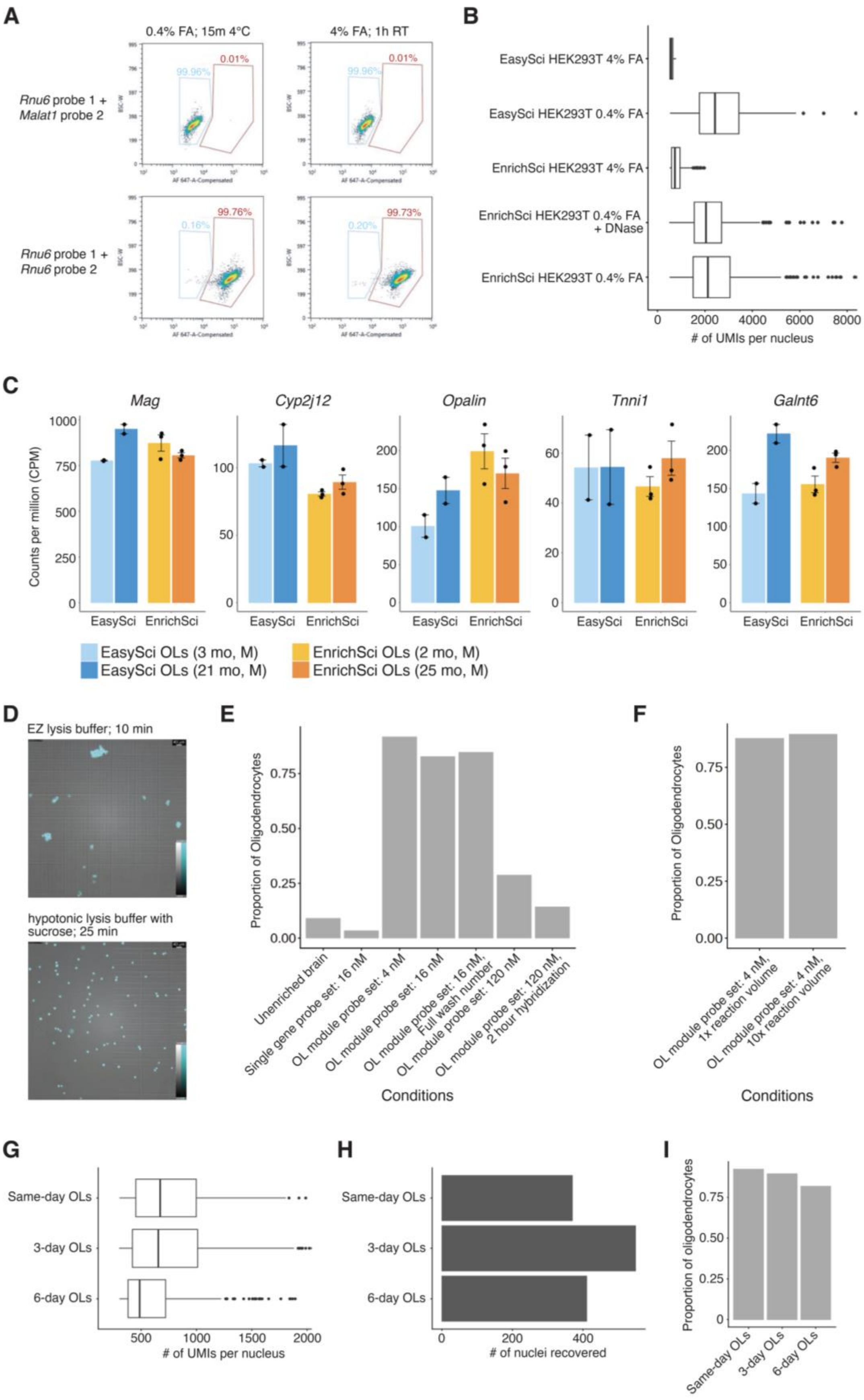

A
0.4% FA; 15m 4°C
4% FA; 1h RT
Rnu6 probe 1 + Malat1 probe 2
Rnu6 probe 1 + Rnu6 probe 2
99.96%
0.01%
0.16%
99.76%
0.20%
99.73%
AF 647-A-Compensated
BSC-W
B
EasySci HEK293T 4% FA
EasySci HEK293T 0.4% FA
EnrichSci HEK293T 4% FA
EnrichSci HEK293T 0.4% FA + DNase
EnrichSci HEK293T 0.4% FA
# of UMIs per nucleus
C
Mag
Cyp2j12
Opalin
Tnni1
Galnt6
Counts per million (CPM)
EasySci
EnrichSci
EasySci OLs (3 mo, M)
EasySci OLs (21 mo, M)
EnrichSci OLs (2 mo, M)
EnrichSci OLs (25 mo, M)
D
EZ lysis buffer; 10 min
hypotonic lysis buffer with sucrose; 25 min
E
Proportion of Oligodendrocytes
Unenriched brain
Single gene probe set: 16 nM
OL module probe set: 4 nM
OL module probe set: 16 nM
OL module probe set: 16 nM, Full wash number
OL module probe set: 120 nM
OL module probe set: 120 nM, 2 hour hybridization
Conditions
F
Proportion of Oligodendrocytes
OL module probe set: 4 nM, 1x reaction volume
OL module probe set: 4 nM, 10x reaction volume
Conditions
G
Same-day OLs
3-day OLs
6-day OLs
# of UMIs per nucleus
H
Same-day OLs
3-day OLs
6-day OLs
# of nuclei recovered
I
Proportion of oligodendrocytes
Same-day OLs
3-day OLs
6-day OLs

Figure S1: EnrichSci was benchmarked and optimized for robust data quality and performance, related to Figure 1. (A) Flow cytometry plots showing HCR signal in HEK293T nuclei fixed in 0.4% (left) versus 4% (right) formaldehyde. Samples were labeled using a negative control mismatched probe pair (top) or a positive control probe pair targeting *Rnu6* (bottom). **(B)** Boxplot showing unique molecular identifier (UMI) count distributions of nuclei profiled by EnrichSci and EasySci, comparing different fixation conditions and the addition of DNase treatment. **(C)** Barplots showing expression of oligodendrocyte-specific module genes (*Mag*, *Cyp2j12*, *Opalin*, *Tnni1*, *Galnt6*) in young and aged male OLs in the EasySci atlas and EnrichSci dataset. Expression is normalized to counts per million (CPM) and shown as mean ± SEM across replicates (dots). **(D)** Comparison of nuclei extraction buffers for A375 cells. Nuclei prepared in a hypotonic lysis buffer with sucrose (bottom) exhibit markedly reduced clumping and higher recovery than those processed with the commercial EZ Lysis Buffer (top). (**E**) Evaluation of HCR RNA FISH conditions for oligodendrocyte enrichment from mouse brain. We varied probe design (single-gene *Sox10* vs. multi-gene oligodendrocyte module probes), probe concentration (4, 16 or 120 nM), wash stringency (standard vs. reduced number of washes) and hybridization time (2 h vs. overnight), and assessed enrichment efficiency by single-nucleus sequencing. The optimal settings—multi-gene probe modules at 4 nM, reduced wash steps, and overnight hybridization—yielded the highest recovery of target cells. (**F**) Comparison of HCR reaction volumes (1× vs. 10×) for large-scale processing of mouse brain nuclei. Both volumes produced comparable enrichment efficiencies. (**G**) Boxplot showing UMI count distributions of nuclei from enriched samples stored at 4°C for 3 and 6 days compared with same-day processing, profiled by EnrichSci. Samples were shallowly sequenced for comparison purposes only (as part of a larger library to ~2,500 reads/nuclei, which is reflected in the low total UMI counts). (**H**) Barplot showing the number of nuclei recovered by EnrichSci from 2 wells loaded at ~666 cells/well under the same storage conditions as (G). **(I)** Barplot showing the proportion of oligodendrocytes in enriched samples under the same storage conditions.

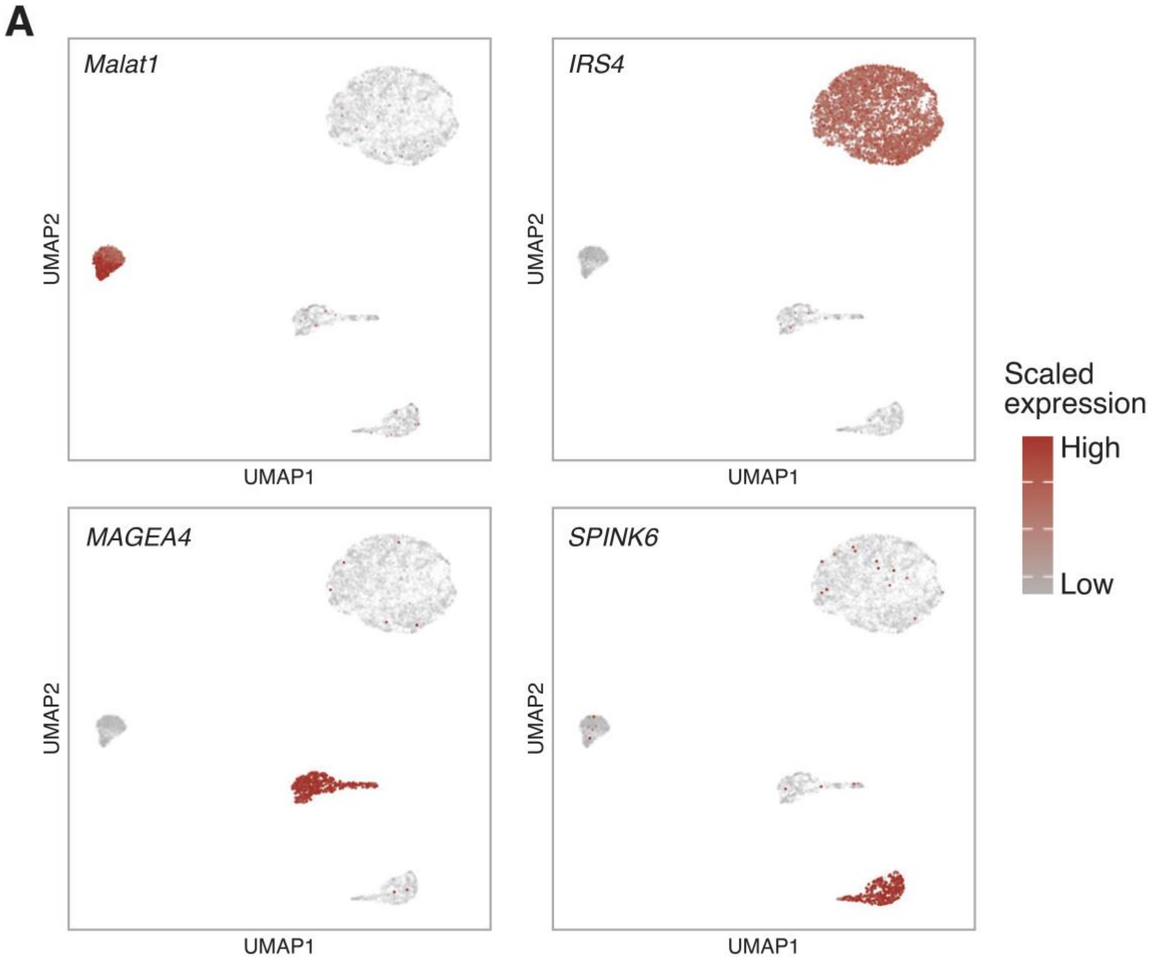

Figure S2: EnrichSci recapitulates the expected molecular states of cell lines profiled in a species-targeted experiment, related to Figure 1. (**A**) UMAP visualization of single-nucleus transcriptomes (n = 9,247) that include individual cell line spike-ins, unenriched mixture, and enriched samples profiled by EnrichSci, colored by the normalized and scaled expression of cell line-specific gene markers.

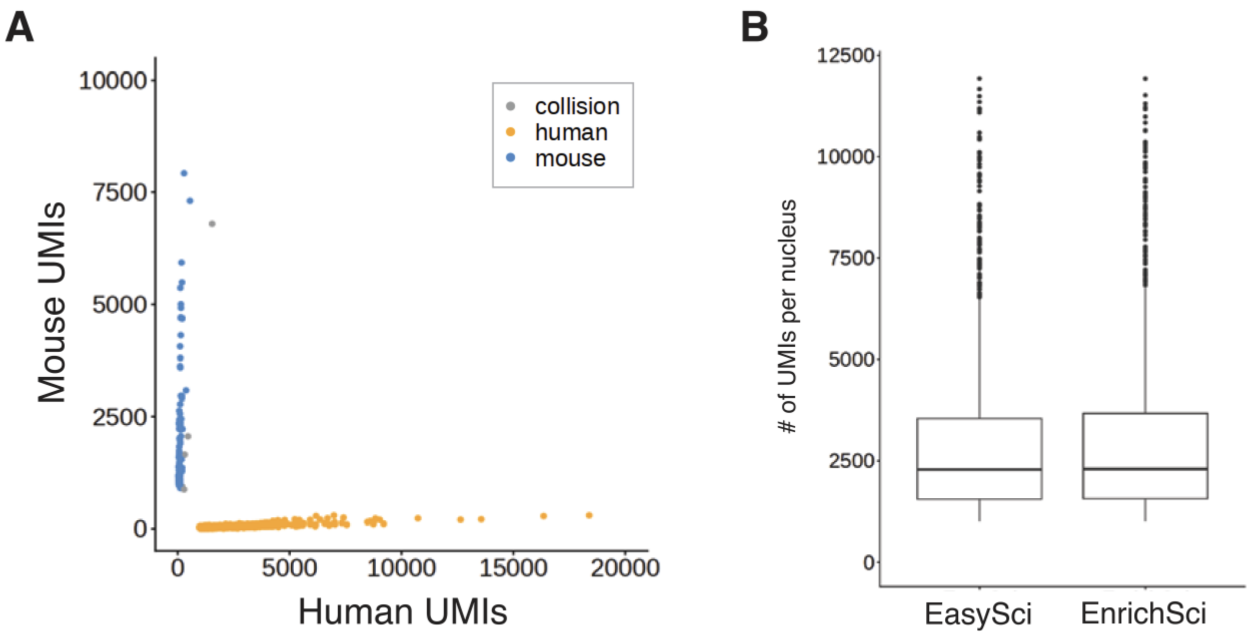

Figure S3: HCR labeling does not compromise single-cell purity or RNA capture efficiency, related to Figure 1. (**A**) Scatter plot of mouse and human UMI counts from the unenriched cell line mixture profiled by EnrichSci. Blue, inferred mouse nuclei (n = 85). Orange, inferred human nuclei (n = 814). Gray, collisions (n = 10). (**B**) Boxplot showing UMI count distribution of nuclei profiled by EasySci (n = 1,535; median = 2,296) and EnrichSci (n = 1,571; median = 2,331).

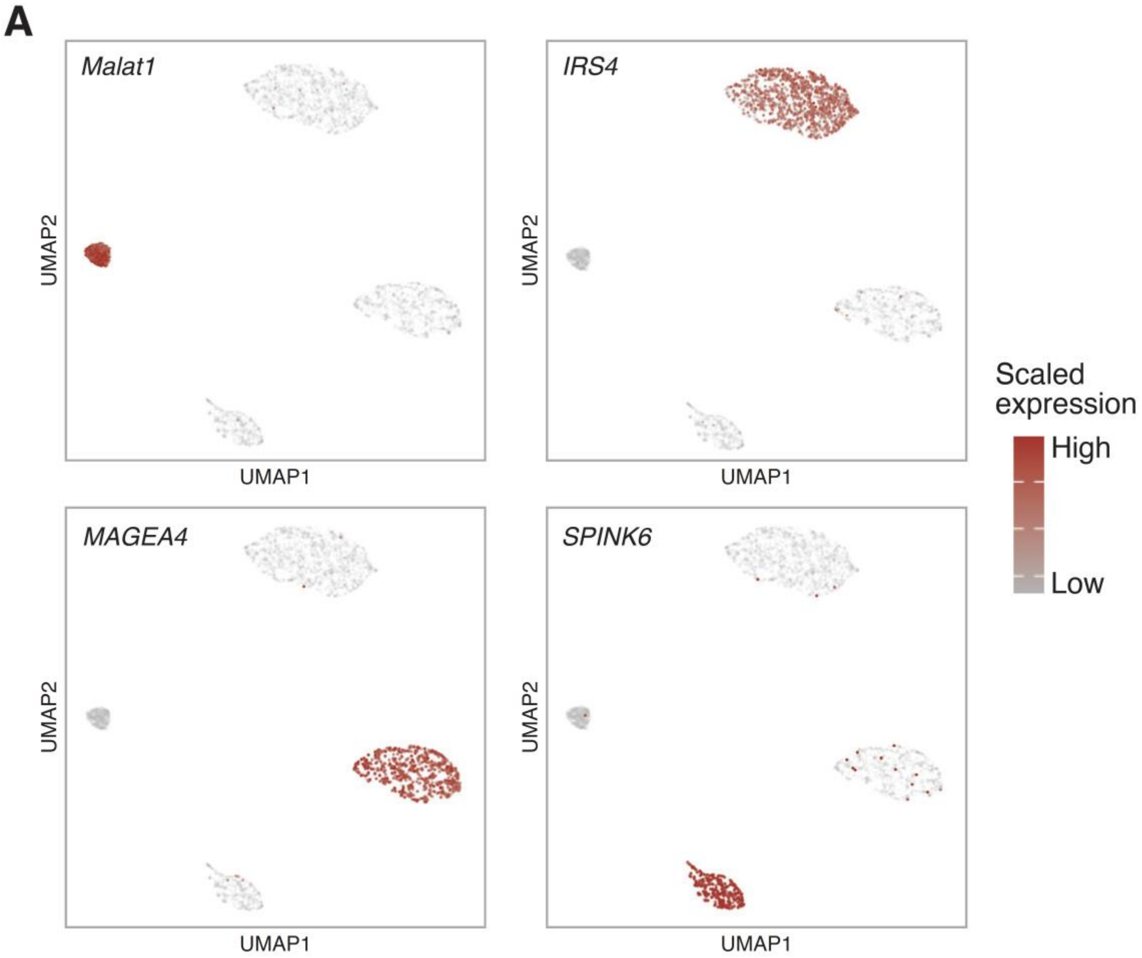

Figure S4: EnrichSci recapitulates the expected molecular states of cell lines profiled in a complex sorting experiment, related to Figure 1. (**A**) UMAP visualization of single-nucleus transcriptomes (n = 4,597) that include individual cell line spike-ins and enriched samples profiled by EnrichSci, colored by the normalized and scaled expression of cell line-specific gene markers.

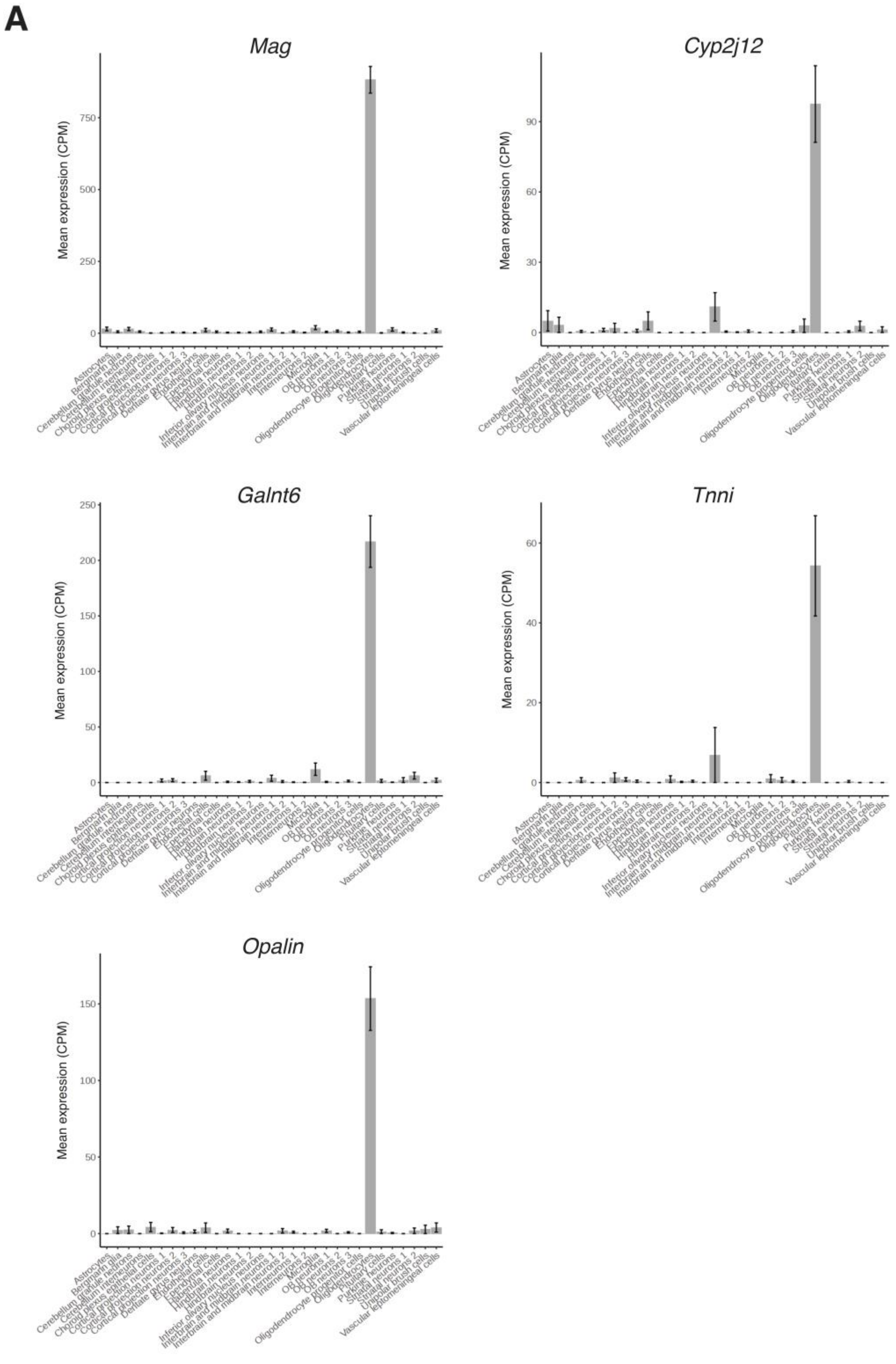

Figure S5: Oligodendrocyte-targeting genes are specifically expressed in the desired cell type, related to Figure 2. (**A**) Barplots showing expression of oligodendrocyte-specific genes across main cell types in the mouse brain.[1] Expression is normalized to counts per million (CPM) and shown as mean ± SEM across replicates.

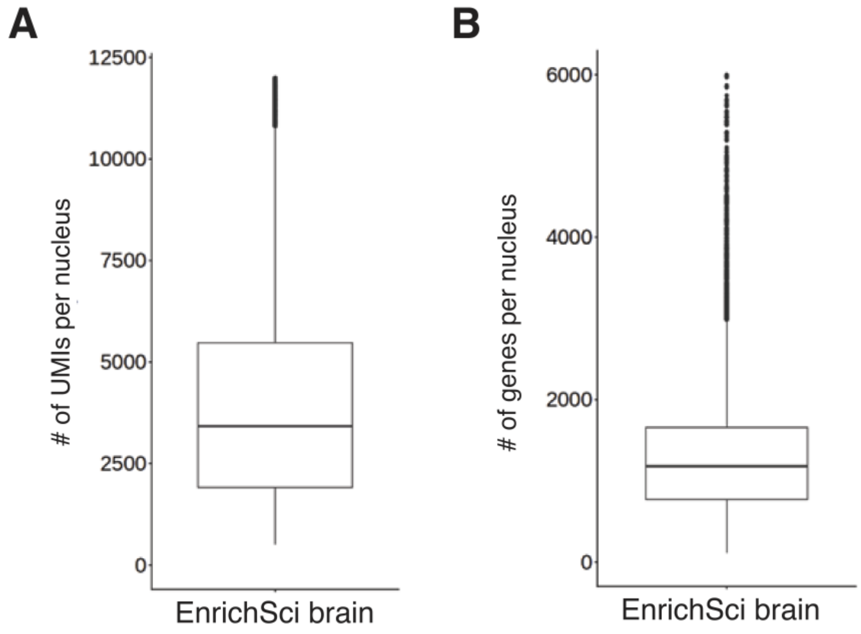

Figure S6: EnrichSci recovered single-nucleus transcriptome profiles of mouse brain nuclei with high signals, related to Figure 2. (**A**) Boxplot showing UMI count distribution of oligodendrocyte-enriched mouse brain nuclei profiled by EnrichSci. (**B**) Boxplot showing gene count distribution of oligodendrocyte-enriched mouse brain nuclei profiled by EnrichSci.

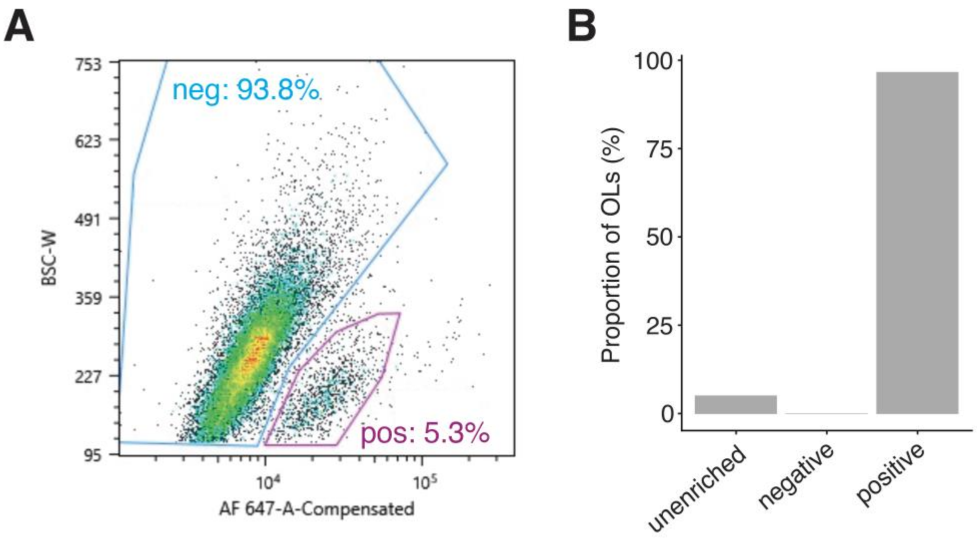

Figure S7: EnrichSci achieves enrichment and complete depletion of oligodendrocytes in positive- and negative-signal populations, related to Figure 2. (**A**) FACS plot showing positive- and negative-signal populations sorted in an oligodendrocyte-targeted experiment performed with fresh HCR reagents. (**B**) Barplot showing the proportion of oligodendrocytes in the populations sorted from (A) and profiled by EnrichSci, compared with an unenriched EasySci control.

A

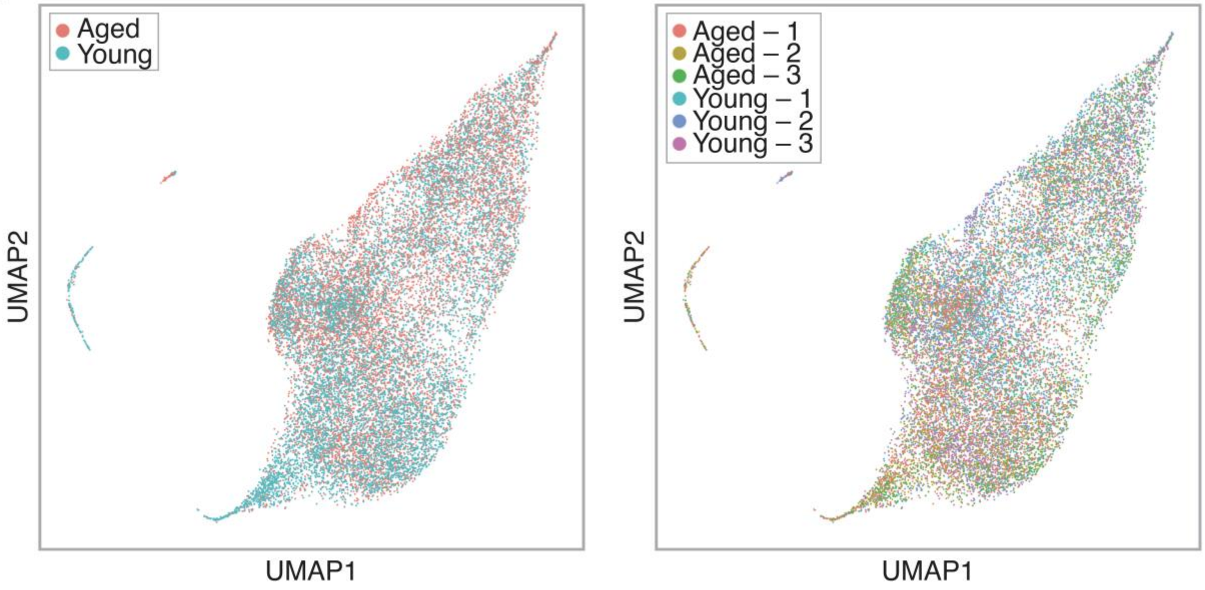

Figure S8: EnrichSci profiles oligodendrocyte nuclei with high sample consistency across age groups and replicates, related to Figure 2. (**A**) UMAP visualization of oligodendrocyte lineage nuclei (n = 18,154) profiled by EnrichSci, colored by age group (left) and individual replicate (right).

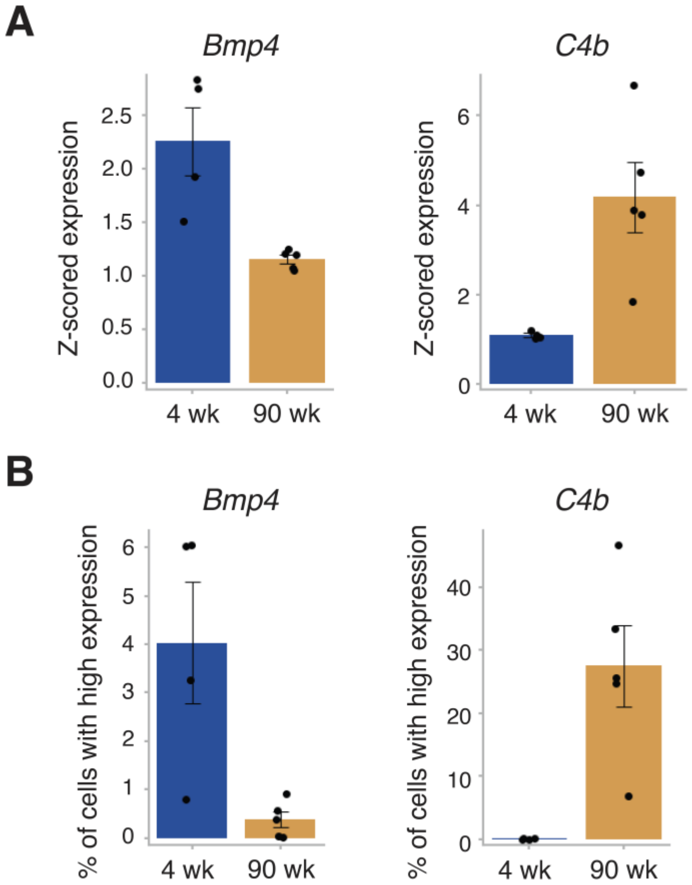

Figure S9: MERFISH confirms aging-associated oligodendrocyte subpopulation changes identified by EnrichSci, related to Figure 2. (A) Barplots showing normalized, log-transformed, and Z-scored expression of COP marker *Bmp4* (left) and reactive OL marker *C4b* (right) in OL lineage cells from young and aged mouse brains, as measured by MERFISH. Bars represent mean ± SEM across replicates (dots). **(B)** Barplots showing the fraction of cells with high expression (Z-score > 2) of *Bmp4* (left) and *C4b* (right) in each condition. Bars represent mean ± SEM across replicates (dots).

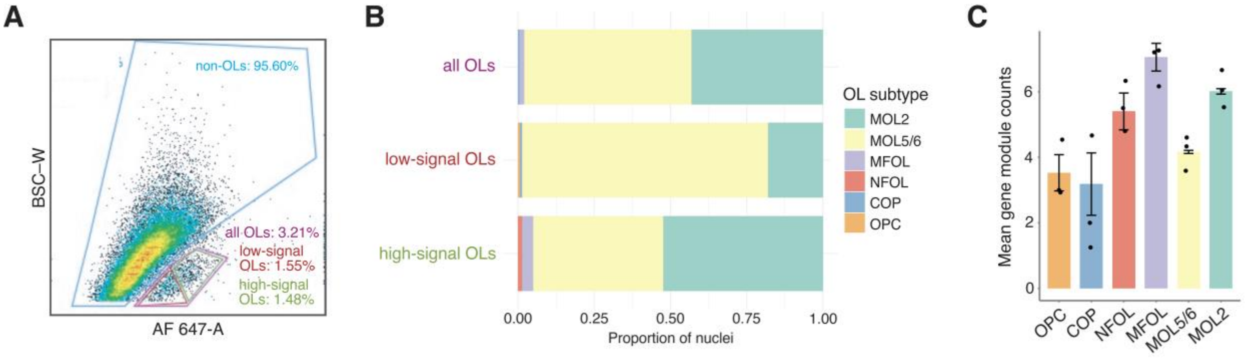

Figure S10: Low- and high-signal oligodendrocytes from complex sorting reflect subtype-specific differences in oligodendrocyte gene module expression, related to Figure 2. (A) FACS plot showing gating of low-signal, high-signal, and all OL populations during complex sorting of OL-enriched aged mouse brain nuclei. **(B)** Barplots showing the OL subtype composition of each population during complex sorting, with bars divided by subtype. **(C)** Barplots showing expression (non-normalized raw counts) of the oligodendrocyte gene module across OL subtypes in the original EnrichSci aged OL data. Bars represent mean ± SEM across replicates (dots).

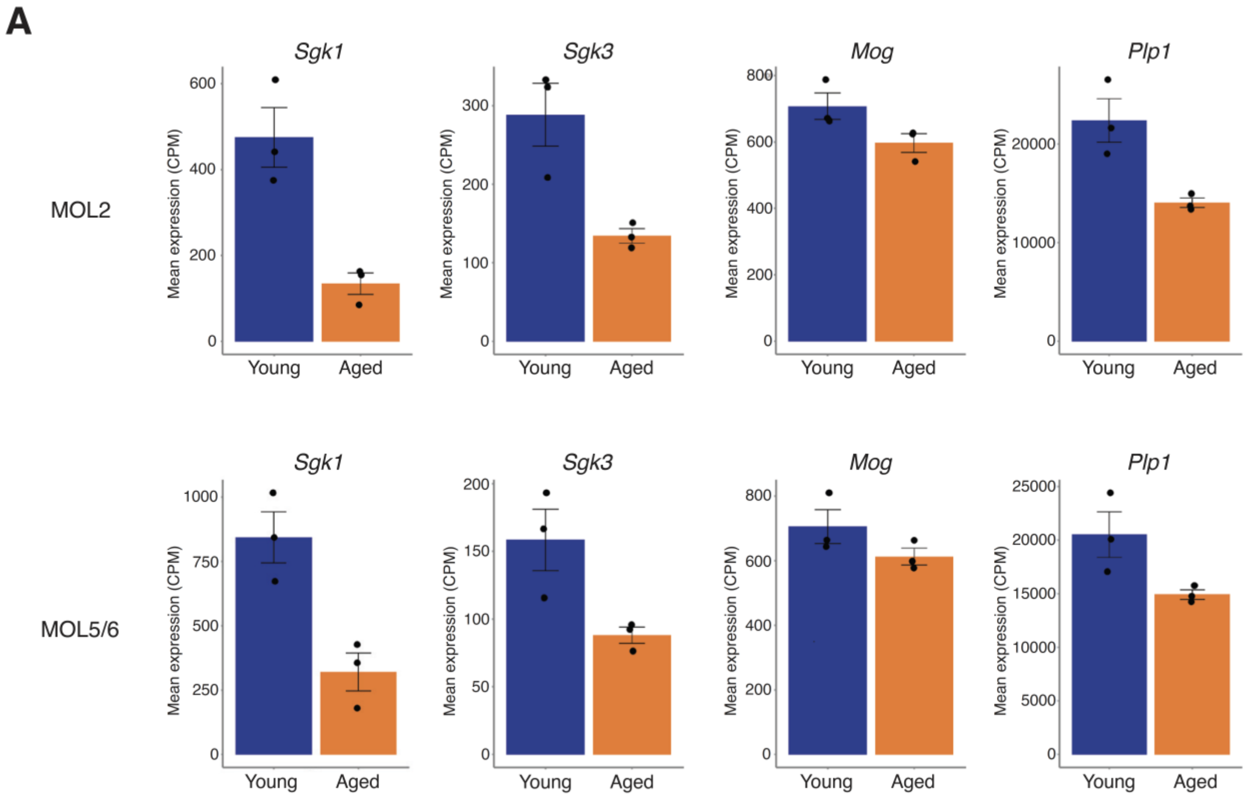

Figure S11: Mature oligodendrocyte subtypes exhibit decreased expression of key oligodendrocyte genes with age, related to Figure 3. (**A**) Barplots showing expression of *Sgk1*, *Sgk3*, *Mog*, and *Plp1* in young and aged MOL2 (top) and MOL5/6 (bottom). Expression is normalized to counts per million (CPM) and shown as mean ± SEM across replicates (dots).

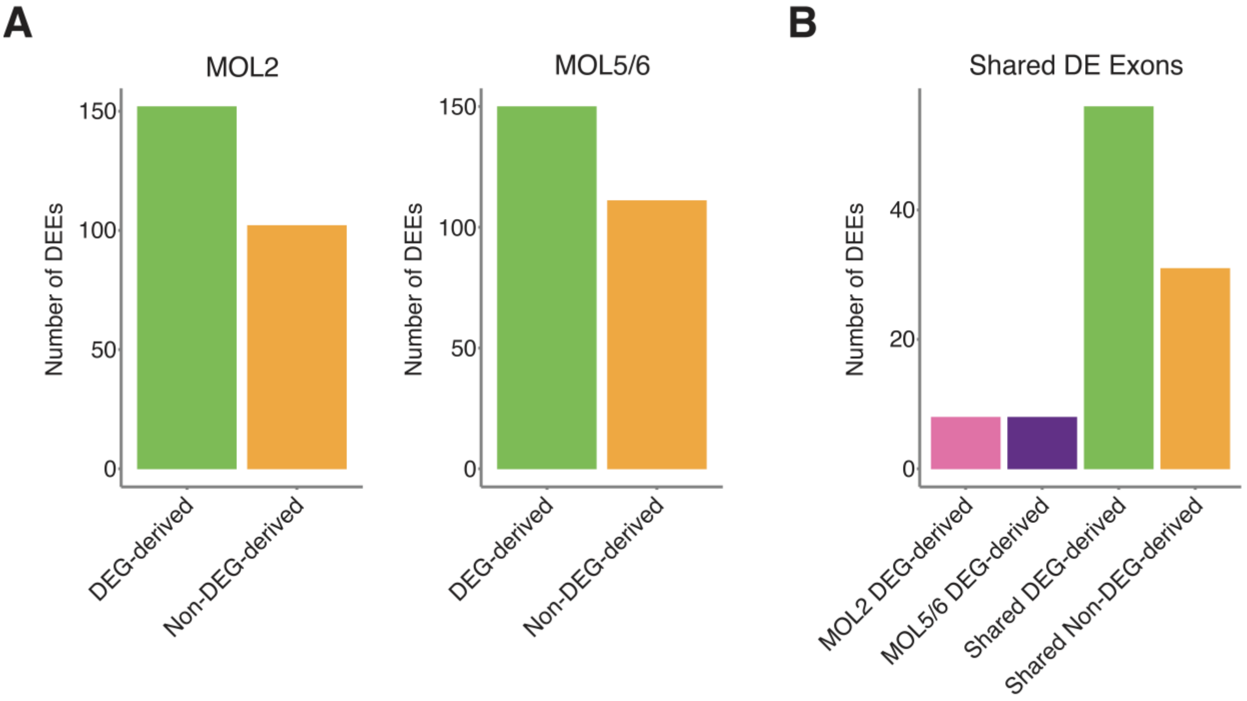

Figure S12: EnrichSci identifies substantial numbers of differentially expressed (DE) exons derived from genes that are not DE, related to Figure 3. (**A**) Barplots showing the number of DE exons (DEEs) that derive from DE genes (DEGs) and non-DEGs in MOL2 (left) and MOL5/6 (right). (**B**) Barplot showing the number of shared DEEs that derive from subtype-specific DEGs, shared DEGs, and shared non-DEGs.

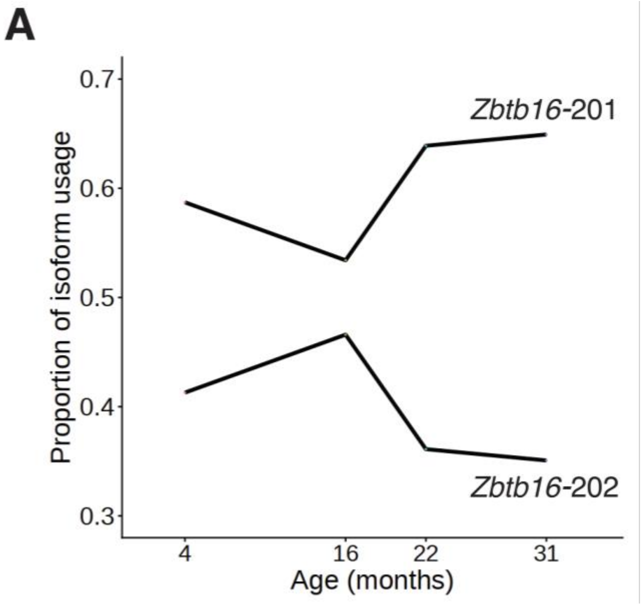

Figure S13: Long-read scRNA-seq identifies age-related shifts in *Zbtb16* isoform usage matching exon 7 changes detected by EnrichSci, related to Figure 3. (**A**) Line plot of isoform expression for *Zbtb16* in male hippocampal oligodendrocytes at 4 ages across the mouse lifespan.

A

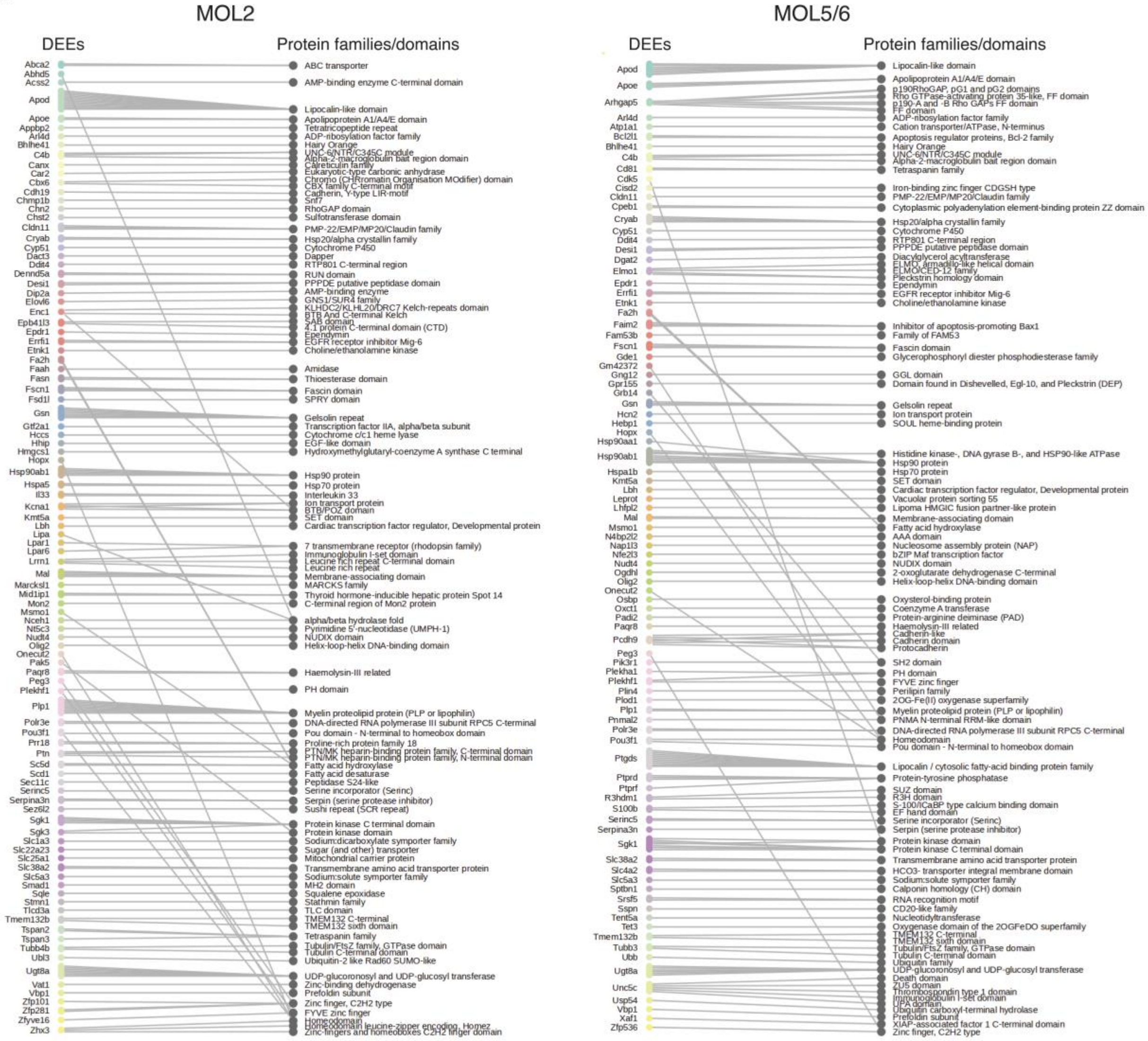

Figure S14: Differentially expressed exons code for diverse functional domains, related to Figure 3. (**A**) Bipartite visualization of DEEs and their mapped protein domains. Each point on the left represents a DEE, grouped and color-coded by parent gene, that maps to at least one annotated Pfam domain. Exons without annotated domains are excluded. Lines connect DEEs to their corresponding protein domains on the right.
